# Spatial conservation prioritisation in data-poor countries: a quantitative sensitivity analysis using multiple taxa

**DOI:** 10.1186/s12898-020-00305-7

**Published:** 2020-06-26

**Authors:** Ahmed El-Gabbas, Francis Gilbert, Carsten F. Dormann

**Affiliations:** 1 Department of Biometry and Environmental System Analysis, University of Freiburg, 79106 Freiburg, Germany; 2 School of Life Sciences, University of Nottingham, Nottingham, UK

**Keywords:** Data-poor countries, Protected areas, Sampling bias, Spatial conservation prioritisation, Species distribution models, Zonation

## Abstract

**Background:**

Spatial conservation prioritisation (SCP) is a set of computational tools designed to support the efficient spatial allocation of priority areas for conservation actions, but it is subject to many sources of uncertainty which should be accounted for during the prioritisation process. We quantified the sensitivity of an SCP application (using software Zonation) to possible sources of uncertainty in data-poor situations, including the use of different surrogate options; correction for sampling bias; how to integrate connectivity; the choice of species distribution modelling (SDM) algorithm; how cells are removed from the landscape; and two methods of assigning weights to species (red-list status or prediction uncertainty). Further, we evaluated the effectiveness of the Egyptian protected areas for conservation, and spatially allocated the top priority sites for further on-the-ground evaluation as potential areas for protected areas expansion.

**Results:**

Focal taxon (butterflies, reptiles, and mammals), sampling bias, connectivity and the choice of SDM algorithm were the most sensitive parameters; collectively these reflect data quality issues. In contrast, cell removal rule and species weights contributed much less to overall variability. Using currently available species data, we found the current effectiveness of Egypt’s protected areas for conserving fauna was low.

**Conclusions:**

For SCP to be useful, there is a lower limit on data quality, requiring data-poor countries to improve sampling strategies and data quality to obtain unbiased data for as many taxa as possible. Since our sensitivity analysis may not generalise, conservation planners should use sensitivity analyses more routinely, particularly relying on more than one combination of SDM algorithm and surrogate group, consider correction for sampling bias, and compare the spatial patterns of predicted priority sites using a variety of settings. The sensitivity of SCP to connectivity parameters means that the responses of each species to habitat loss are important knowledge gaps.

## Background

Biological diversity is declining, with many species facing extinction in response to multiple interacting anthropogenic factors [1–5]. Protected areas (PAs) represent a core response strategy, currently covering about 15% of the global land area [6]. However, PAs are often bedevilled by lack of resources and trained personnel, isolation, and poor management [4, 7–9]. They are declared on expert opinion or on aesthetic value, neither of which is likely to ensure biodiversity persistence [10–12]. As a consequence of political opportunity rather than conservation assessment, many PAs are biased towards remote, uneconomic and low-cost areas, regardless of their biodiversity value [13, 14].

The Convention on Biological Diversity (CBD) agreed a *Strategic Plan for Biodiversity 2011*–*2020*, including the Aichi targets [15], two of which address threatened species (target 12) and an expansion of the global PA network to cover at least 17% of terrestrial land and 10% of coastal and marine areas by 2020 (target 11). Implementing these requires substantial effort to avoid creating PAs that exist purely as legal fictions (‘paper parks’: [16]). PAs should be ecologically representative, effectively managed, well connected, and based on sound science to include biodiversity and ecosystem services [15].

A spatially explicit suitability assessment for PAs needs to consider at least the following elements: (1) indicator taxa; (2) distribution data; and (3) connectivity of sites to facilitate movement. Ecologically coherent PAs aim to maximise representation and persistence of a wide range of habitats, ecosystems and species [3, 10, 13]. Since it is impossible to conserve all species and habitats simultaneously, limited conservation resources should be strategically and effectively prioritised [3].

Spatial conservation prioritisation (SCP) uses computational tools to prioritise areas for conservation actions [13]. It requires comprehensive, high-resolution, up-to-date data on the distribution of biodiversity, but such data are usually incomplete or do not exist at all, making it challenging to develop reliable assessments in data-poor countries [1, 8]. Such analyses are frequently performed using biodiversity surrogates, i.e. well-studied taxa representing biodiversity as a whole [13, 17]. Surrogates vary in their representativeness [3, 7, 18], and their choice is more often driven by data availability than ecological appropriateness [19].

Potential distribution maps from species distribution models (SDMs) are frequently used in conservation planning to overcome the lack of adequate surveys [20]. SDMs are statistical models that relate data on species presence (and absence) to the environment to predict potential distribution [21]. Despite their computational convenience, SDMs are subject to multiple sources of uncertainty [20, 22]: location, detectability, spatial autocorrelation, algorithm, and environmental predictors. Spatial sampling bias inherent in opportunistic presence-only data (the typical case in data-poor situations) can also lead to biased estimates of suitability [23], for example towards easily accessible areas [24]. Methods to correct for sampling bias (e.g. [23, 25]) should be considered in planning [26].

Landscape connectivity influences species persistence through dispersal, source-sink dynamics, colonisation and gene flow [27]. Maintaining connectivity between PAs improves their ecological functioning and is now considered pivotal for effective planning [28–30]. Well-connected PAs facilitate the movement of widespread species and gene flow among patchily distributed populations, improving persistence under future land-use and climate change [18]. If connectivity is not included, SCP can prioritise isolated, fragmented, and small sites insufficient for long-term persistence in fragmented landscapes.

Applying any SCP software involves decisions on settings, which inevitably affect the results [3, 26, 31, 32]. We assess the sensitivity of SCP in data-poor situations (represented by Egypt) to common sources of uncertainty. We quantify variability in the results of Zonation to distributional data quality and user-defined Zonation parameters, including: (1) biodiversity surrogate (butterflies, reptiles, mammals, or all three groups together); (2) SDM algorithm (Maxent *vs* elastic net); (3) with *vs* without weighting by prediction uncertainty; (4) with *vs* without bias correction; (5) different options for integrating connectivity; (6) weighting species by Red-List status; (7) different rules of priority; and (8) accounting for existing PAs *vs* a clean-slate situation. Although changing any of these factors will change the results, we assess here which dominate. We then evaluate the effectiveness of the current PA network in the conservation of the Egyptian fauna, and then map important areas for future PA expansions in Egypt.

## Methods

### Study species and data

The main source of the presence data was the database collated by the BioMAP project (Biodiversity Monitoring and Assessment Project, 2004–2008; *Butterflies:* [33], *Reptiles:* [34], *Mammals:* [35]), with revisions and additions from subsequent fieldwork and literature (see Additional file 1: Appendix S1). We excluded 76 species with few records (< 8 unique pixels and < 5 spatial blocks), as they make spatial-block cross-validation (see below) impossible. The final species list comprises a total of 178 species (32 butterfly, 75 reptile, and 71 mammal species; Additional file 1: Table S1).

### SDMs & sampling bias

We used two SDM algorithms to model potential distributions: Maxent (ver. 3.3.3 k: [36]) and elastic net ([37]; implementing a down-weighted Poisson regression), both under the point-process modelling framework [38]. As the focus of this study is the evaluation of Zonation, we only give an overview of the SDM-approach here (details in [39]). Models were calibrated using fivefold spatial-block cross-validation, resulting in a mean prediction for each species. Uncertainty in the prediction (predictive consistency) was computed as the average spatial congruence (Schoener’s; [40]) for all ten possible pairs of cross-validated predictions; this served as a weight in Zonation (see below).

We assessed how much correcting for sampling bias changed the results by supplying Zonation with two sets of prediction maps, without correction (models fitted using only environmental variables), and with correction (using model-based bias correction: [25]). In the latter, SDMs were fitted including a bias layer of accessibility variables (distances to closest roads, cities, and PAs) and adjusting predictions to a constant accessibility of zero distance (for more details see: [25, 39]).

### SCP & potential sources of uncertainty

We used Zonation (version 4.0; [2, 41]) to prioritise the Egyptian landscape for conservation. The Zonation outputs include a priority rank map and performance curves. The former represents a nested hierarchy of conservation value (top 5% cells are within the top 10%, etc.), showing the priority areas [41]. Zonation starts with the full landscape, and then iteratively removes the cells with the smallest marginal loss of conservation value (subject to species-specific weights, cost, connectivity, etc.). Performance curves demonstrate the quality of the solution by quantifying the fraction of the original distribution of each species (or their average) retains in response to successive removals of cells from the landscape ([42]; see Additional file 1: Figure S1 for a performance curve example).

Zonation uses different rules to calculate the marginal loss of conservation value and therefore the order of cell removal: additive-benefit function (ABF) and core-area zonation (CAZ) emphasise different but complementary aspects of biodiversity [41]. ABF emphasises species richness and is more appropriate for surrogates; CAZ emphasises species rarity by maintaining high-quality locations regardless of the local richness, and is more appropriate for the actual conservation targets [42].

To be more realistic, conservation planning should include the cost of implementation [43, 44], but obtaining cost data is frequently challenging [3]. No land prices (acquisition cost) are available across Egypt, and hence we constrained Zonation always to give low priority to agricultural and urban areas (Additional file 1: Figure S2), heavily modified and settled since Pharaonic times [45].

As different species do not have equal conservation importance, some species can be given a higher weight, influencing the order of cell removal [42]. We used two weighting methods: Red-List status and predictive consistency. We assigned weights between 1 and 5 according to national Red List assessments (*Butterflies:* [33], *Reptiles:* [34], *Mammals:* [35]; Additional file 1: Table S1). We compared equal-weighted vs Red List-weighted solutions. We used predictive consistency as described above as a second weighting option. Mean spatial congruence ranges from zero (very high predictive uncertainty) to one (identical predictions, no uncertainty). We used both weighting schemes factorially, yielding four options of species weighting.

Zonation offers different ways of integrating connectivity, such as boundary length penalty [28], distribution smoothing [2], and boundary-quality penalty (BQP; [30]). We used BQP, which requires a user-defined species-specific response to fragmentation (habitat loss) in the neighbour cells, represented by two parameters (radius and response curve). The radius defines the number of effective neighbour cells (~ distance) whose degradation or loss will affect the local cell value. The response curve describes the degree to which habitat loss in neighbour cells affects the local population [30]. Values are typically determined from habitat models [41] or expert ecological knowledge (e.g. [30, 46, 47]). Since both parameters are hard to obtain accurately, we used combinations of three response curves (low, moderate, and high effect; Additional file 1: Figure S3) and three radii (1–3 cells) plus no connectivity, making ten options in total.

Finally, we allowed for two possibilities with the current PA network. First, we ignored it, allowing Zonation to prioritise any area of Egypt. Second, we forced Zonation always to retain the cells of the current PA network as the highest priority cells in Egypt.

In total, we conducted 2560 Zonation analyses: *four* surrogate groups (butterflies, reptiles, mammals and all three together) × *two* modelling algorithms (Maxent, elastic net) × *two* biases (without, with sampling-bias correction) × *two* cell-removal rules (ABF, CAZ) × *four* weighting schemes (none, Red-List, predictive uncertainty, and both) × *ten* connectivity options × *two* PA-integration options (without, with PA masking). An example reproducible R-code used to generate the input files for zonation can be found in this link: https://github.com/elgabbas/Conservation-Prioritisation-Sensitivity. To quantify the main drivers of uncertainty in Zonation’s output, we used the mean species representation (= average proportion of remaining distribution of species) in the top 17% priority cells as the response variable, estimated from performance curves. This value denotes the target area to be protected in Egypt by 2020. A *randomForest* analysis quantified the permutation importance of these factors. We also estimated the most important first-order interactions among factors using the mean sum of squares from a generalised linear model.

### Gap analysis and potential areas for PA expansion

To assess the effectiveness of Egypt’s PA network, we concentrated on Maxent SDM because it fitted slightly better on cross-validation than elastic net, using species weights from Red-List status and predictive consistency so as to give high weight to threatened species with high predictive consistency. To restrict the number of possibilities, we used an average connectivity (line 3 in Additional file 1: Figure S3) with the radius set at two neighbour cells and the CAZ cell-removal rule to retain high-quality core areas for conservation. Where necessary, we make comparisons between the outputs for Maxent vs elastic net and CAZ vs ABF.

To conduct the gap analysis, we first allowed Zonation to prioritise the Egyptian landscape without considering the positions of the existing PAs. We then used three ways to assess the current PAs: first, we measured the point-biserial correlation between the (continuous) priority rank and the binary allocation of each pixel either to a PA or to non-PA land; second, we quantified the spatial overlap between current PAs and top-priority sites of equal area using Jaccard’s similarity index; and third, by calculating Kendall’s correlation between the priority rank and the proportion of cells protected at each 1% rank intervals (following [48]). We expect PAs with good spatial allocation to have high values for biserial correlation, Jaccard’s index, and Kendall’s correlation. We identified top-priority areas outside PAs (candidate locations for PA expansion) by forcing PAs to have the highest priority and then determining the best locations required to expand to 17% of the Egypt’s area.

## Results

**Fig. 1:**
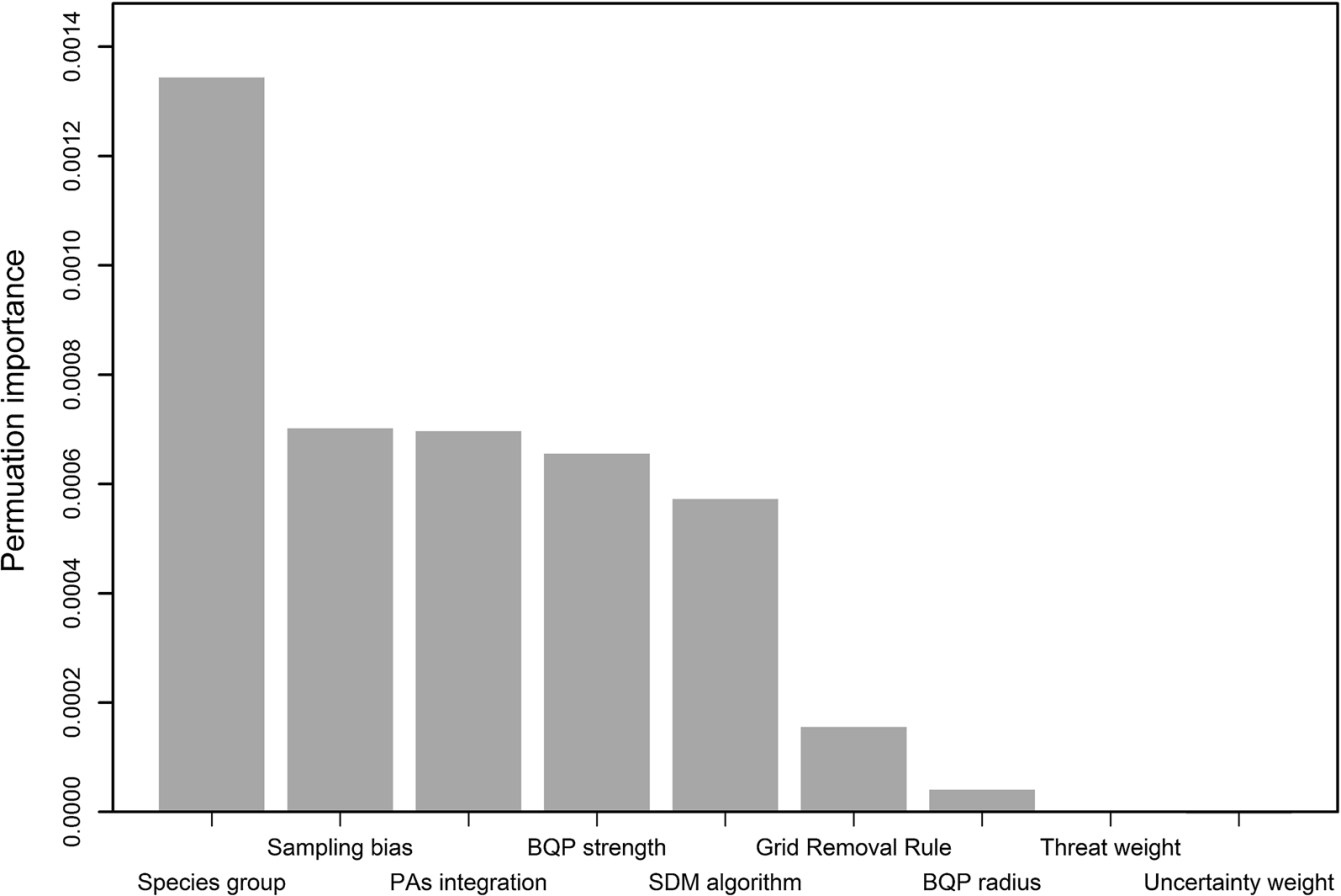
Permutation importance of factors affecting Zonation sensitivity across 2560 option combinations (randomForest model). The dependent variable is the mean species representation in the top 17% priority cells. (For the top 10% and 25%, see Additional file 1: Figure S5.) Statistical interactions are included in the measure of variable importance

**Fig. 2:**
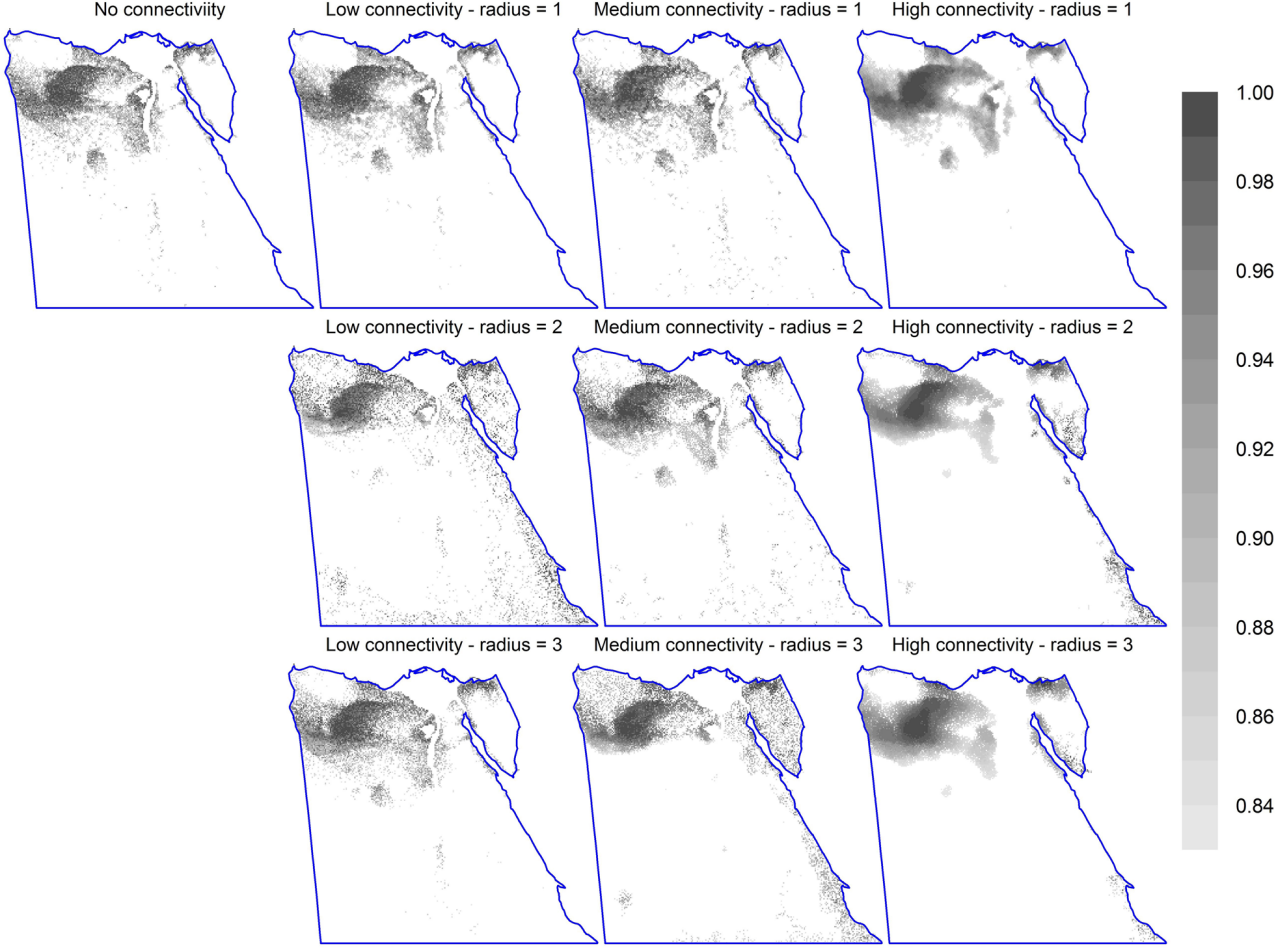
The spatial distribution of the top 17% priority cells for different connectivity options (using options CAZ, bias-free predictions, all study species, Maxent). The top-left map shows the pattern of important sites without connectivity integration. The second to fourth columns are for equivalent maps with steeper response curves (low, medium, and high connectivity; curves 2-4 in Additional file 1: Figure S3, respectively); while rows are for different numbers of effective neighbour cells (1 to 3). (Equivalent maps using ABF are shown in Additional file 1: Figure S4.)

The main factor affecting the sensitivity of prioritisation (Fig. 1) was the choice of surrogate group, followed by correction for sampling bias, the strength of connectivity (response curve), and the choice of SDM algorithm. Correction for sampling bias led to higher species representation. The spatial pattern of the top-priority cells depended on how strong the dependence of local populations in each cell was on habitat loss in the neighbourhood. The steeper the response curve (high dependency), the more clumped and the less scattered the solution. This effect was more pronounced for CAZ than for ABF (compare Fig. 2 and Additional file 1: Figure S4).

Maxent models led to higher species representation than elastic net. The different cell-removal rules, the radius of effective neighbour cells (connectivity), and weighting species by Red-List status or predictive consistency did not affect sensitivity much (Fig. 1); however, using ABF or weighting species by their Red-List status led to relatively lower species representation. The use of different landscape threshold percentages (10%, 17%, 25%) did not affect the overall relative importance; however, the choice of the surrogate group had lower importance when the top 10% priority of the landscape was used (Additional file 1: Figure S5).

The most important interactions were as follows. Between sampling-bias correction and SDM algorithm, correction for sampling bias led to higher improvement for elastic net. Between the surrogate group and sampling-bias correction, correction led to greater improvement for mammals than for reptiles. Between the surrogate group and SDM algorithm, Maxent had higher species representation for butterflies and mammals (Additional file 1: Figure S6).

We expected (by design) that incorporating the current PAs would lead to different spatial allocation of important sites than solutions that did not account for PAs. Indeed, PA incorporation was one of the most important factors affecting prioritisation (Fig. 1). The interactions between incorporating PAs and other important factors (sampling bias correction and the choice of SDM algorithm and surrogate group) were also significant (Additional file 1: Figure S6).

**Fig. 3:**
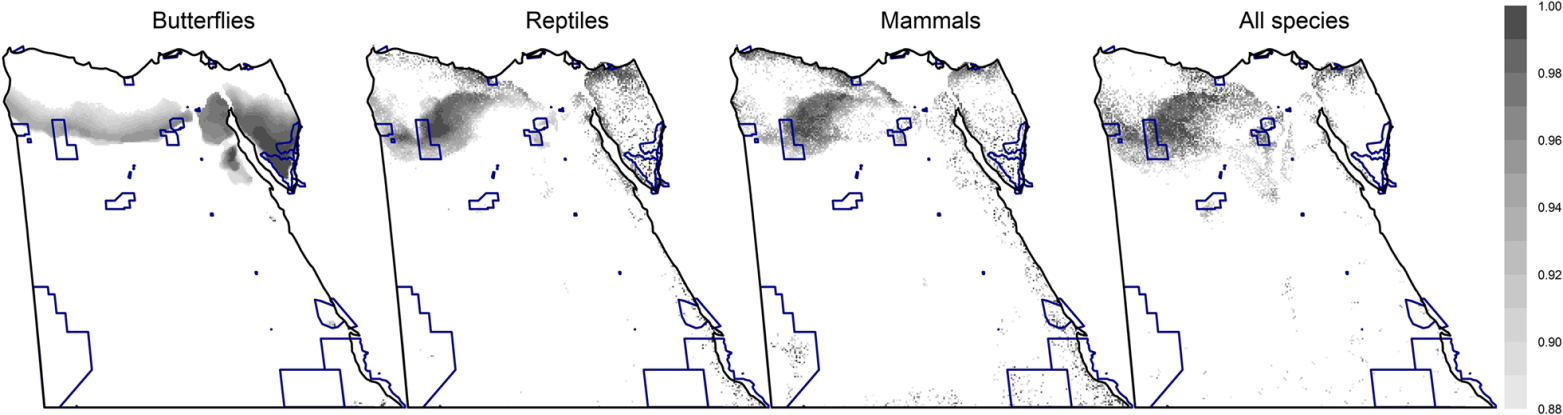
The spatial distribution of the top 12% priority cells (the darker, the higher the priority) for the four surrogates using core-area zonation, overlaid with current protected areas in Egypt (blue borders) (using bias-free predictions from Maxent). Equivalent maps using additive-benefit function are shown in Additional file 1: Figure S7

**Fig. 4:**
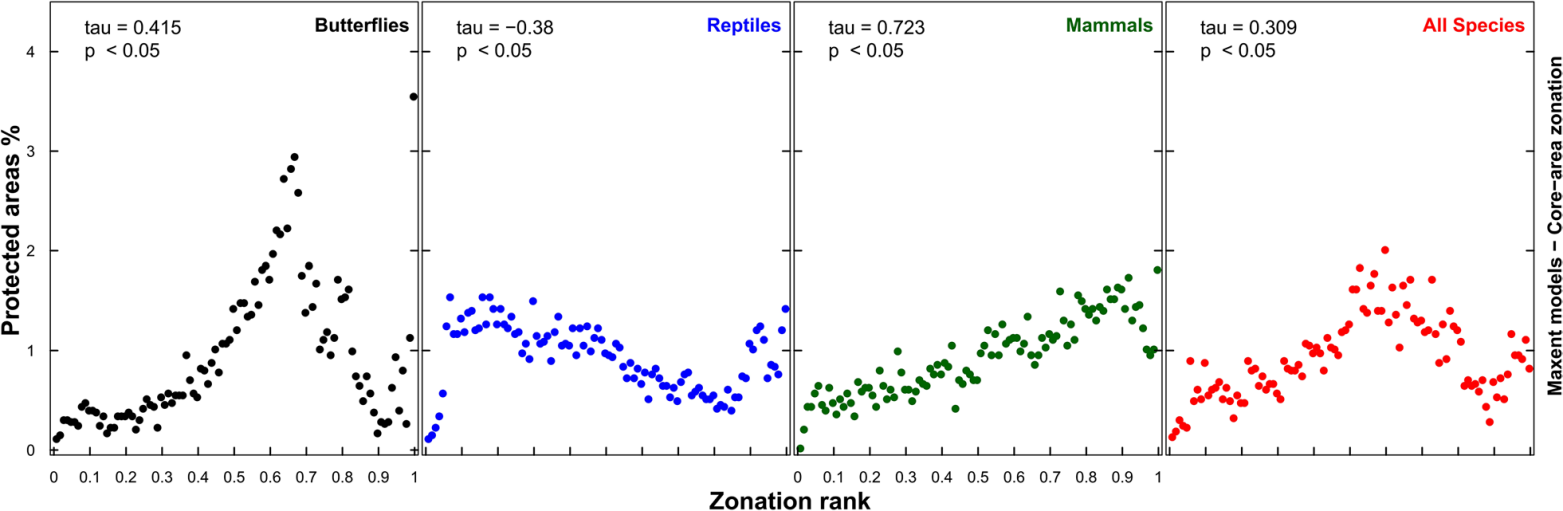
The fraction of cells protected per Zonation rank for different surrogates using core-area zonation (using bias-free predictions from Maxent). The number on each panel represents Kendall’s correlation coefficient. (For equivalent results using additive-benefit function or elastic net, see Additional file 1: Figures S8, S9)

When the Egyptian PAs were not incorporated, the spatial allocation of the top-priority cells varied depending on the choice of taxon, with the majority of cells outside current PAs (78–90% under CAZ, similar for ABF: Fig. 3 and Additional file 1: Figure S7). In other words, the overlap of priority cells with current PAs (Jaccard’s index) was very low (4.8–9.8%) and independent of the choice of the surrogate taxon, sampling-bias correction and connectivity. The correlation between current PAs and the overall priority ranking in Egypt was very weak (− 0.11 to 0.14). There was a fair to good positive Kendall’s correlation between cell ranking and the fraction of cells protected at each 1% rank interval for mammals (τ = 0.72), butterflies (0.42), and all-species solutions (0.31), but a negative correlation for reptiles (− 0.38; all for CAZ; see Fig. 4). When ABF was used, negative correlations occurred for all taxa (from − 0.57 to − 0.29) except butterflies (0.4; see Additional file 1: Figure S8). Similar results were found for elastic net, with non-significant correlations for reptiles and mammals for CAZ (Additional file 1: Figure S9).

**Fig. 5:**
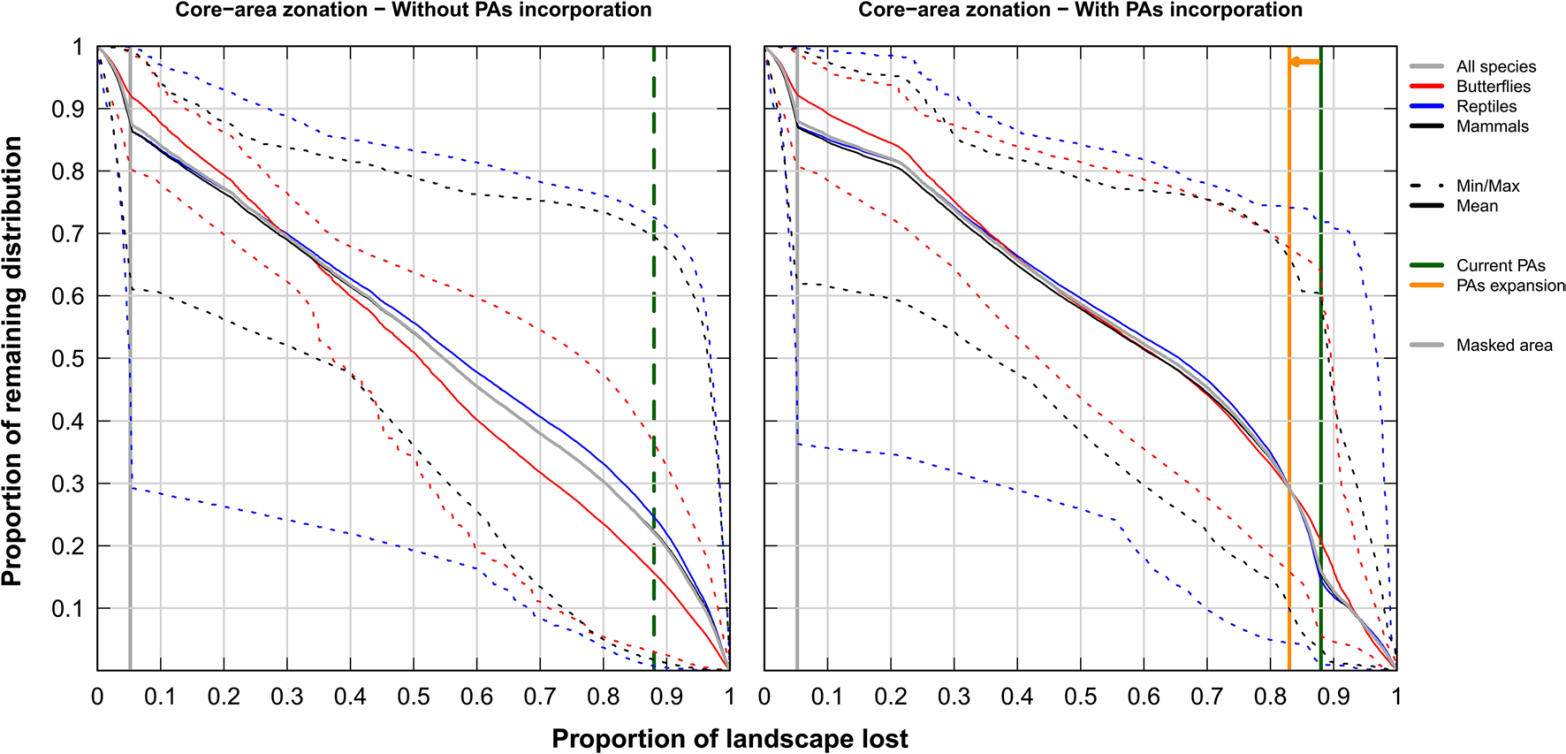
Performance curves for Zonation analyses (Maxent, CAZ, all species together). Left panel is without PA integration. Solid curves represent the average performance curve for all species or per species-group; while dashed lines represent the overall minimum and maximum performance curve per species group. The vertical grey line is for urban and agricultural areas (Additional file 1: Figure S2) forced to have low priority value; while the dashed vertical green line represents top priority sites existent in an area equals to the area covered by PAs. The right panel represents equivalent analysis with Egyptian PAs forced to have the highest priority scores. The vertical green line represents the area covered by PAs; while the vertical orange line represents the proposed areas for PAs expansion to 17% of Egypt. Performance curves are described in Additional file 1: Figure S1 and in the main text. For equivalent curves using ABF, see Additional file 1: Figure S10. Results for elastic net are shown in Additional file 1: Figure S11

Performance curves showed that the top-priority sites equivalent in area to the current PAs retained an average species representation of about 22% (all-species analyses; Fig. 5, left panel), compared with only about 15% retained within the current PAs (Fig. 5, right panel). Results are similar for CAZ and ABF, with slightly higher values for elastic net (Fig. 5 and Additional file 1: Figures S10, S11). For the separate taxa, top-priority sites again maintained a higher mean species representation (40% for butterflies; 35% for reptiles, and 33% for mammals). Urban and agricultural areas retained an average species representation of about 13% of all study species (Fig. 5 and Additional file 1: Figures S10, S11), suggesting that although these areas are not crucial for species protection, they still have conservation importance for those species that depend on humans, either directly or via agriculture (e.g. rodents and some butterflies).

**Fig. 6:**
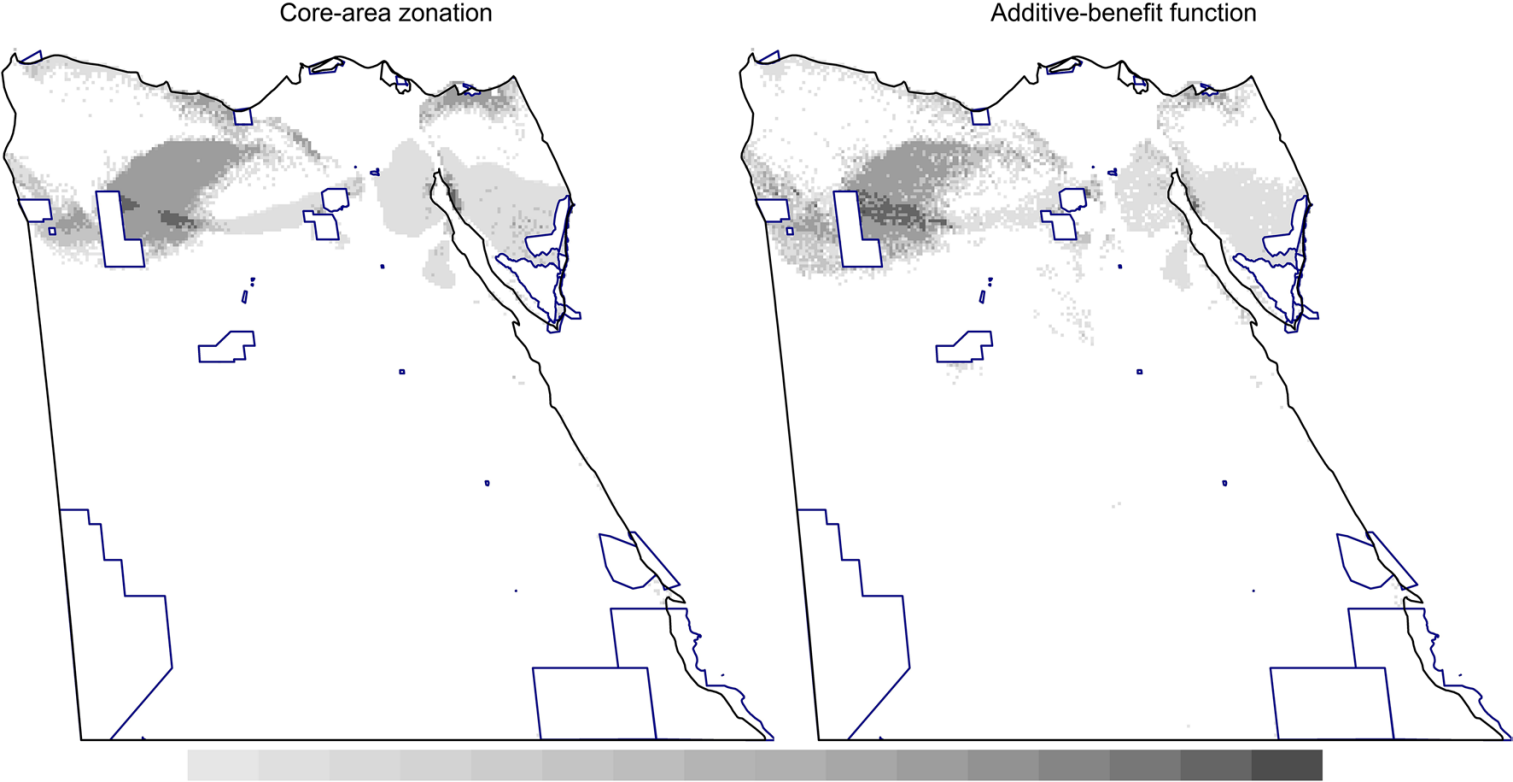
The overall pattern of top priority sites using CAZ (left) or ABF (right), both using bias-free predictions from Maxent. Each map shows the summed rankings of the top 17% sites from the four surrogates used. Shading within current PAs (blue borders) are not shown to highlight the pattern for PA expansion (darker shading indicates higher rankings). The pattern for each species group is shown in Additional file 1: Figure S12. For equivalent maps using elastic net, see Additional file 1: Figure S13

When PAs were forced into the solution, expanding current PAs to 17% of Egypt almost doubled the average species representation (all-species: Fig. 5 and Additional file 1: Figures S10, S11), with relatively higher values for elastic net. The pattern of top-priority sites varied slightly among species groups (Additional file 1: Figure S12), which together require more than 17% of the Egyptian landscape to conserve all three species groups simultaneously. We summarised the overall pattern of the top-priority sites by adding together the rankings of the top 17% cells of the four surrogate options (Fig. 6 and Additional file 1: Figure S13). Potential areas for PAs expansion include the Qattara Depression westwards to include the surroundings of the Siwa Oasis, the Mediterranean Coast west of El-Omayed, the Wadi El-Natrun area southwards, a coastal strip of North Sinai, the majority of central Sinai, coastal areas on both sides of the Suez Canal and the Gulf of Suez (including El-Galala mountains), inland wadis west of Hurghada, and the periphery of Wadi El-Gimal PAs (Fig. 6, Additional file 1: Figures S13, S15).

## Discussion

### Drivers of conservation prioritisation sensitivity

Target areas for conservation should maximise species representation and their long-term persistence for best use of limited resources. SCP is a method of identifying priority areas and assessing the effectiveness of reserves. However, its efficient use is hampered by uncertainties and the paucity of good-quality data. Many procedural decisions influence the robustness of the assessments, and therefore conservation practitioners should account for possible sources of uncertainty during SCP analyses [3, 26, 31, 32]. In this study, we quantified the sensitivity of prioritisation by varying user decisions experimentally. The main sources of uncertainty were the choice of surrogate group, sampling bias, the strength of the BQP response curve (i.e. the connectivity), and the choice of SDM algorithm, which collectively reflect our incomplete knowledge of species ecology and distribution.

Conservation planning is sensitive to the choice of taxon used as surrogate for biodiversity [19]. Here, we used four surrogates for the Egyptian fauna. Although their priority rankings are (fairly) positively correlated (Additional file 1: Table S2), choosing one strongly affects the mean species representation in the solution (Fig. 1), implying that they are not adequate to represent each other [7, 17]. As a consequence, even the collective use of all three will not adequately represent Egyptian biodiversity as a whole. The vertebrate groups only partially cover for butterflies, implying that data on other taxa is essential for comprehensive conservation planning in Egypt. Such data are currently not available for the majority of taxa, and hence improving sampling and data-sharing in data-poor countries are prerequisites to making SCP analyses more representative [19].

The issue of data quality also becomes apparent in the effect of sampling bias on SDM predictive performance. We used model-based bias correction to improve predictions for presence-only SDMs [25, 39, 49], and sampling bias was the second most important factor affecting prioritisation. Correction led to a different spatial allocation of important sites and greater representation of species. Accordingly, we used ‘bias-free’ predictions in the final assessments. The use of biased data for conservation planning identifies suboptimal conservation sites, which are weakly correlated with ideal reserves, leading to the requirement of more area for conserving fewer species [50]. Sampling-bias correction should be used for conservation planning in data-poor situations or when there are clear signs of bias in the available data. However, correction for sampling bias is not always successful [23, 39], and hence the improvement of data quality should be a priority.

Maintaining connectivity among conservation sites is important for long-term conservation [9]. However, the effective integration of connectivity into SCP requires knowledge of home range, dispersal ability and sensitivity to habitat fragmentation of each species. This information is onerous to estimate correctly [31] and is unavailable for most species, especially for cryptic species and in data-poor countries. Connectivity methods that do not require information on species ecology are possible. One example is the boundary-length penalty [28], but this is a generic (not ecology-driven) method that maintains site aggregation via assigning a high penalty to solutions that have a high edge-to-area ratio, and hence does not account for habitat quality near the border [41].

Connectivity response curves strongly affected the spatial distribution of the top-priority sites (Fig. 2 and Additional file 1: Figure S4), and hence connectivity demands careful attention in SCP analyses. Our use of the low-connectivity response curve (line 2 in Additional file 1: Figure S3) did not lead to a different outcome compared to analyses which did not consider connectivity at all (line 1 in Additional file 1: Figure S3). In contrast, the use of medium or high response curves (lines 3 and 4 in Additional file 1: Figure S3) led to more aggregated top-priority sites (Fig. 2 and Additional file 1: Figure S4) and a lower mean species representation [46]. Maintaining connectivity between important sites leads to the inclusion of sites of low importance in the solution, and hence involves trade-offs [29, 31]. On the other hand, the radius of effective neighbour cells was not of much importance. As aggregated important sites are a safer investment than fragmented or isolated solutions [31], we conditioned our final analyses on a medium level of connectivity (line 3 in Additional file 1: Figure S3). However, this is not the ideal approach of integrating connectivity into Zonation when species differ in their sensitivity to habitat loss. The lack of relevant information for the Egyptian species is a common situation in data-poor countries.

Although Maxent and elastic net had very similar performances when used under the point-process modelling framework [39, 49], their use here contributes to the sensitivity of prioritisation. Predictions from Maxent led to a higher mean species representation than elastic net. Similarly, Lentini and Wintle [19] found that the spatial allocation of priority sites was highly dependent on the SDM algorithm used. More than one SDM algorithm should probably be used, comparing the spatial pattern of their priority sites. However, the effect of modelling algorithm had little effect on the proposed sites for PA expansion (Fig. 6 and Additional file 1: Figure S13).

The use of CAZ or ABF cell removal rules led to some variation in prioritisation. Each rule identified different top-priority sites, especially for reptiles and mammals (Fig. 3 vs Additional file 1: Figure S7). This effect was expected, because the calculation of the marginal loss of conservation value is different in both rules: CAZ emphasises species rarity, while ABF emphasises species richness (for details, see [41]). Their differences here can be attributed partially to the effect of connectivity, which affects CAZ more than ABF (Fig. 2 vs Additional file 1: Figure S4); [51]. The areas proposed for PA expansion were not too different whichever cell removal rule was used (Fig. 6 and Additional file 1: Figures S12, S13).

Weighting species by Red-List status was less important than the other factors described above, but it led to smaller mean species representation. Weighting species includes trade-offs, because species assigned higher weights will have a higher representation in the solution at the expense of other species [47]. In contrast, weighting by predictive consistency was not important, probably because spatial-block cross-validation produced high and not very variable values (median = 0.8; Additional file 1: Figure S14).

### The effectiveness of current Egyptian PAs and areas for potential expansion

Since 1983, thirty Egyptian PAs have been declared, covering approximately 14.6% of the total area of Egypt (Additional file 1: Figure S15), gazetted after fieldwork, literature reviews and recommendations of experts [45, 52]. Although the surveyed sites show good spatial coverage (Additional file 1: Figure S16), they are inevitably incomplete and are taxonomically biased towards charismatic or well-known taxa.

Ideally the majority of top-priority sites would be located inside PAs, but the high permutation importance of PA incorporation indicates the suboptimality of current Egyptian PAs. The performance curve for the top-priority sites irrespective of PAs is more concave (Fig. 5 left) than for current PAs (Fig. 5 right), meaning that the cells with the highest priority across Egypt have higher species representation than sites within PAs. Current PAs are reasonably correlated with the important sites for butterflies and when all species were used together, but only very weakly or even negatively for reptiles and mammals. The correlation between priority rankings across Egypt and the PAs was similarly very weak in all cases, as was the intersection between current PAs and top-priority sites (Jaccard’s index) for all taxa, cell-removal rules, and SDMs. Thus modelling shows that though slightly better for butterflies, the PA network does not cover species-rich areas for mammals well, and is also inadequate for reptiles in all scenarios.

Previous studies evaluating the effectiveness of the Egyptian PAs have shown contrasting results. Newbold et al. [45] suggested that they are more effective for butterflies and mammals than random sites, containing higher than average species richness, and similar results were found for medicinal plants [53, 54]. However, simple species richness as an indicator is sensitive to sampling bias [55], fails to consider complementarity, and ignores important threatened species living in less-rich areas [56] and unique assemblages [5]. Therefore the use of species richness alone for conservation planning may create solutions that are too optimistic. In contrast, Zonation is a complementarity-based method, designed to ensure that important sites maximise species representation.

Effective PAs should be resilient to climate change [57]. We did not consider climate change here so as to simplify the analysis of prioritisation sensitivity. Using Egyptian reptiles, the current PA network has a significantly higher Zonation ranking than unprotected areas, but the difference declines under climate change [34]. To prevent species loss under climate change, new PAs in Egypt are probably required for effective conservation in the future [34, 54, 58]. For example, the Suez Gulf coast, Qattara Depression and Red Sea Coast are important for medicinal plants under climate change [54]. These areas, along with the Gebel El-Galala area, Mediterranean Coast, and Central to North Sinai, are important for reptiles under climate change [34]. These assessments suggest areas similar to those proposed for PA expansion here (Fig. 6 and Additional file 1: Figure S13).

It is interesting that, despite the large uncertainty explored in this study via the 2560 SCP analyses, there is surprising congruence in the spatial distribution of the priority sites chosen by Zonation across all options. The frequency with which cells were chosen to be within the highest or lowest set of priority sites across all options is shown in Additional file 1: Figure S17. This encourages us to feel the final recommendations may be rather robust to the modelled uncertainties.

The proper evaluation of Egypt’s PAs is in its infancy, and our analysis is inevitably incomplete. Our results should not be considered definitive, because of the unknown level of error and uncertainty accompanying species distribution data, distribution modelling and the prioritisation process. Conservation planning is sensitive to commission errors in predicted distribution maps (false presences—conserving areas lacking the species of interest; [24]). Although we attempted to minimise this error using spatial-block cross-validation, predicted distributions still have an unknown level of error. The use of only a single type of species response to habitat loss can also be criticised. We cannot guarantee that conserving the top-priority areas identified here will ensure long-term species persistence, because this requires explicit population modelling, such as population viability analysis [20, 28]. Some priority areas outside PAs are under great pressure from human uses (e.g. tourism on the Mediterranean and the Red-Sea coasts; mining in the Qattara Depression).

The actual conservation value of the top-priority sites requires fine-scale field validation [3] to verify their suitability. Such validation must be undertaken by the Egyptian Nature Conservation Sector when planning for PA expansion. Likewise, our evidence for the low conservation efficiency of the current PA network should *not* encourage decision-makers in Egypt to alter current PAs until intensive good-quality data on a range of taxa become available. This will require much time and effort. Our aspirations for an expansion of the PA network should not distract from the urgent need for improving management within current PAs.

Data quality greatly affects conservation planning [50]. Despite the endeavours of Egypt’s Nature Conservation Sector, many more initiatives are required to improve inventories within and outside PAs, and also effective ecosystem management within PAs. A national scheme for biodiversity data collection and sharing would greatly facilitate future conservation planning. Due to the limitations of currently available data, the effectiveness of the PAs will need to be re-evaluated using improved inventory data on a wider range of taxa (e.g. birds, amphibians, plants, invertebrates, etc.), and include climate change. Data on additional species would make the SCP analysis more robust and stable and better represents the overall Egyptian biodiversity [59]. Future evaluation should be run in close partnership with scientists, experts on Egyptian biodiversity, decision-makers, stakeholders and local communities to minimise the gap between doing research and its implementation [20, 57].

## Conclusion

The robustness of conservation planning applications is subject to many sources of uncertainty which should be accounted for during the prioritisation process. Conservation planning in data-poor situations is sensitive to the selection of the surrogate group, correction for sampling bias, connectivity parameters, and the choice of modelling algorithm; collectively, these reflect data quality issues. This underlines the urgent need to improve data quality in the data-poor countries to enhance the usefulness of SDMs and conservation planning applications for long-term biodiversity conservation. We recommend the use of data on as many species groups as possible and more than one modelling algorithm to obtain a robust and stable conservation planning. Sampling bias can highly affect the efficiency of SCP output and therefore should be corrected for. Maintaining connectivity between top priority sites is essential for the effective long-term conservation of many species and therefore should be carefully integrated into conservation planning. However, as species-specific responses to habitat loss represents an important knowledge gap. We highlight the need for studies elaborating the response to habitat loss for less-studied species. Using currently available data on the Egyptian butterflies, reptiles, and mammals, the Egyptian protected areas network seems to be inefficient for wildlife conservation. We determined the top priority sites for further on-the-ground field evaluation as potential areas for protected areas expansion.

## Supplementary information

**Additional file 1.** Additional Tables, Figure and Appendix.

## Data Availability

The dataset analysed during the current study is presented in Additional file 1: Appendix S1.

## Abbreviations

ABF: Additive-benefit function
BQP: Boundary-quality penalty
CAZ: Core-area zonation
CBD: The Convention on Biological Diversity
PAs: Protected areas
SCP: Spatial conservation prioritisation
SDMs: Species distribution models
