# Supplementary material for: Spatial conservation prioritisation in data-poor countries: a quantitative sensitivity analysis using multiple taxa
Source: BMC Ecol. 2020 Jun 26;20:35. doi: 10.1186/s12898-020-00305-7 (PMC7318458; doi:10.1186/s12898-020-00305-7)
Supplement: Supplementary file 1 — Additional file 1. Additional Tables, Figure and Appendix. [file 12898_2020_305_MOESM1_ESM.docx]

*Supporting Information for “****Spatial conservation prioritisation in data-poor countries: a quantitative sensitivity analysis using multiple taxa****”* *– BMC Ecology*

*Ahmed El-Gabbas^1*^, Francis Gilbert^2^, Carsten F. Dormann^1^*

*^1^ Department of Biometry and Environmental System Analysis, University of Freiburg, D-79106 Freiburg, Germany*

*^2^ School of Life Sciences, University of Nottingham, Nottingham, United Kingdom*

*∗ To whom correspondence should be addressed; E-mail:* *elgabbas@outlook.com*

**Table S1:** List of species used or excluded in this study. **RL** represents per-species national Red List status; while **W** represent the assigned weight

***(A)*** *Study species*

|  | ***Species*** | **RL** | **W** |
| --- | --- | --- | --- |
|  | **Butterflies** | | |
| 1 | *Agrodiaetus loewii* | VU | 3 |
| 2 | *Apharitis acamas* | VU | 3 |
| 3 | *Azanus ubaldus* | LC | 1 |
| 4 | *Belenois aurota* | NA | 1 |
| 5 | *Borbo borbonica* | NA | 1 |
| 6 | *Catopsilia florella* | NA | 1 |
| 7 | *Chilades trochylus* | LC | 1 |
| 8 | *Colias croceus* | NA | 1 |
| 9 | *Colotis fausta* | VU | 3 |
| 10 | *Danaus chrysippus* | LC | 1 |
| 11 | *Deudorix livia* | LC | 1 |
| 12 | *Euchloe aegyptiaca* | EN | 4 |
| 13 | *Euchloe belemia* | DD | 1 |
| 14 | *Gegenes nostrodamus* | LC | 1 |
| 15 | *Hypolimnas misippus* | NA | 1 |
| 16 | *Iolana alfierii* | LC | 1 |
| 17 | *Junonia hierta* | LC | 1 |
| 18 | *Lampides boeticus* | LC | 1 |
| 19 | *Leptotes pirithous* | LC | 1 |
| 20 | *Lycaena phlaeas* | NA | 1 |
| 21 | *Melitaea deserticola* | VU | 3 |
| 22 | *Papilio saharae* | VU | 3 |
| 23 | *Pelopidas thrax* | LC | 1 |
| 24 | *Pieris rapae* | LC | 1 |
| 25 | *Plebejus philbyi* | VU | 3 |
| 26 | *Pontia daplidice* | LC | 1 |
| 27 | *Pontia glauconome* | LC | 1 |
| 28 | *Spialia doris* | LC | 1 |
| 29 | *Tarucus rosaceus* | LC | 1 |
| 30 | *Vanessa atalanta* | NA | 1 |
| 31 | *Vanessa cardui* | NA | 1 |
| 32 | *Zizeeria karsandra* | LC | 1 |
|  | | | |
|  | **Reptiles** | | |
| 33 | *Acanthodactylus aegyptius* | LC | 1 |
| 34 | *Acanthodactylus boskianus* | LC | 1 |
| 35 | *Acanthodactylus longipes* | VU | 3 |
| 36 | *Acanthodactylus pardalis* | VU | 3 |
| 37 | *Acanthodactylus scutellatus* | LC | 1 |
| 38 | *Agama spinosa* | LC | 1 |
| 39 | *Cerastes cerastes* | LC | 1 |
| 40 | *Cerastes vipera* | LC | 1 |
| 41 | *Chalcides cf. humilis* | LC | 1 |
| 42 | *Chalcides ocellatus* | LC | 1 |
| 43 | *Chamaeleo africanus* | EN | 4 |
| 44 | *Chamaeleo chamaeleon* | LC | 1 |
| 45 | *Cyrtopodion scabrum* | LC | 1 |
| 46 | *Echis coloratus* | LC | 1 |
| 47 | *Echis pyramidum* | LC | 1 |
| 48 | *Eirenis coronella* | VU | 3 |
| 49 | *Eryx colubrinus* | VU | 3 |
| 50 | *Eryx jaculus* | LC | 1 |
| 51 | *Eumeces schneiderii* | LC | 1 |
| 52 | *Hemidactylus flaviviridis* | VU | 3 |
| 53 | *Hemidactylus robustus* | VU | 3 |
| 54 | *Hemidactylus turcicus* | LC | 1 |
| 55 | *Laudakia stellio* | LC | 1 |
| 56 | *Leptotyphlops cairi* | EN | 4 |
| 57 | *Lytorhynchus diadema* | LC | 1 |
| 58 | *Macroprotodon cucullatus* | VU | 3 |
| 59 | *Malpolon moilensis* | LC | 1 |
| 60 | *Malpolon monspessulanus* | LC | 1 |
| 61 | *Mesalina bahaeldini* | VU | 3 |
| 62 | *Mesalina guttulata* | LC | 1 |
| 63 | *Mesalina olivieri* | LC | 1 |
| 64 | *Mesalina pasteuri* | VU | 3 |
| 65 | *Mesalina rubropunctata* | LC | 1 |
| 66 | *Naja haje* | LC | 1 |
| 67 | *Naja nubiae* | VU | 3 |
| 68 | *Natrix tessellata* | VU | 3 |
| 69 | *Ophisops occidentalis* | VU | 3 |
| 70 | *Platyceps florulentus* | LC | 1 |
| 71 | *Platyceps rogersi* | LC | 1 |
| 72 | *Platyceps saharicus* | LC | 1 |
| 73 | *Pristurus flavipunctatus* | VU | 3 |
| 74 | *Psammophis aegyptius* | LC | 1 |
| 75 | *Psammophis schokari* | LC | 1 |
| 76 | *Psammophis sibilans* | LC | 1 |
| 77 | *Pseudotrapelus sinaitus* | LC | 1 |
| 78 | *Ptyodactylus guttatus* | LC | 1 |
| 79 | *Ptyodactylus hasselquistii* | LC | 1 |
| 80 | *Ptyodactylus siphonorhina* | LC | 1 |
| 81 | *Ramphotyphlops braminus* | NA | 1 |
| 82 | *Scincus scincus* | LC | 1 |
| 83 | *Spalerosophis diadema* | LC | 1 |
| 84 | *Sphenops sepsoides* | LC | 1 |
| 85 | *Stenodactylus mauritanicus* | VU | 3 |
| 86 | *Stenodactylus petrii* | LC | 1 |
| 87 | *Stenodactylus sthenodactylus* | LC | 1 |
| 88 | *Tarentola annularis* | LC | 1 |
| 89 | *Tarentola mauritanica* | LC | 1 |
| 90 | *Tarentola mindiae* | VU | 3 |
| 91 | *Telescopus dhara* | LC | 1 |
| 92 | *Testudo kleinmanni* | VU | 3 |
| 93 | *Trachylepis quinquetaeniata* | LC | 1 |
| 94 | *Trachylepis vittata* | VU | 3 |
| 95 | *Trapelus mutabilis* | LC | 1 |
| 96 | *Trapelus pallidus* | LC | 1 |
| 97 | *Trapelus savignii* | VU | 3 |
| 98 | *Tropiocolotes bisharicus* | VU | 3 |
| 99 | *Tropiocolotes nattereri* | LC | 1 |
| 100 | *Tropiocolotes steudneri* | LC | 1 |
| 101 | *Tropiocolotes tripolitanus* | LC | 1 |
| 102 | *Uromastyx aegyptia* | LC | 1 |
| 103 | *Uromastyx ocellata* | EN | 4 |
| 104 | *Uromastyx ornata* | VU | 3 |
| 105 | *Varanus griseus* | LC | 1 |
| 106 | *Varanus niloticus* | VU | 3 |
| 107 | *Walterinnesia aegyptia* | VU | 3 |
|  | | | |
|  | **Mammals** | | |
| 108 | *Hemiechinus auritus* | LC | 1 |
| 109 | *Paraechinus aethiopicus* | LC | 1 |
| 110 | *Crocidura olivieri* | VU | 3 |
| 111 | *Rousettus aegyptiacus* | LC | 1 |
| 112 | *Taphozous nudiventris* | VU | 3 |
| 113 | *Taphozous perforatus* | LC | 1 |
| 114 | *Asellia tridens* | LC | 1 |
| 115 | *Rhinolophus clivosus* | LC | 1 |
| 116 | *Rhinolophus mehelyi* | EN | 4 |
| 117 | *Rhinopoma cystops* | LC | 1 |
| 118 | *Rhinopoma microphyllum* | VU | 3 |
| 119 | *Tadarida aegyptiaca* | VU | 3 |
| 120 | *Tadarida teniotis* | VU | 3 |
| 121 | *Nycteris thebaica* | LC | 1 |
| 122 | *Eptesicus bottae* | VU | 3 |
| 123 | *Hypsugo ariel* | VU | 3 |
| 124 | *Otonycteris hemprichii* | LC | 1 |
| 125 | *Pipistrellus kuhlii* | LC | 1 |
| 126 | *Pipistrellus rueppellii* | VU | 3 |
| 127 | *Plecotus christii* | LC | 1 |
| 128 | *Lepus capensis* | LC | 1 |
| 129 | *Allactaga tetradactyla* | EN | 4 |
| 130 | *Jaculus jaculus* | LC | 1 |
| 131 | *Jaculus orientalis* | VU | 3 |
| 132 | *Eliomys melanurus* | EN | 4 |
| 133 | *Acomys cahirinus* | LC | 1 |
| 134 | *Acomys dimidiatus* | LC | 1 |
| 135 | *Acomys russatus* | LC | 1 |
| 136 | *Arvicanthis niloticus* | LC | 1 |
| 137 | *Dipodillus campestris* | VU | 3 |
| 138 | *Dipodillus dasyurus* | LC | 1 |
| 139 | *Dipodillus mackilligini* | VU | 3 |
| 140 | *Dipodillus simoni* | VU | 3 |
| 141 | *Gerbillus amoenus* | LC | 1 |
| 142 | *Gerbillus andersoni* | VU | 3 |
| 143 | *Gerbillus floweri* | VU | 3 |
| 144 | *Gerbillus gerbillus* | LC | 1 |
| 145 | *Gerbillus henleyi* | LC | 1 |
| 146 | *Gerbillus perpallidus* | LC | 1 |
| 147 | *Gerbillus pyramidum* | LC | 1 |
| 148 | *Meriones crassus* | LC | 1 |
| 149 | *Meriones libycus* | LC | 1 |
| 150 | *Meriones shawi* | EN | 4 |
| 151 | *Mus musculus* | NA | 1 |
| 152 | *Nesokia indica* | EN | 4 |
| 153 | *Pachyuromys duprasi* | VU | 3 |
| 154 | *Psammomys obesus* | LC | 1 |
| 155 | *Rattus norvegicus* | NA | 1 |
| 156 | *Rattus rattus* | NA | 1 |
| 157 | *Sekeetamys calurus* | LC | 1 |
| 158 | *Spalax aegyptiacus* | EN | 4 |
| 159 | *Canis aureus* | DD | 1 |
| 160 | *Vulpes rueppellii* | LC | 1 |
| 161 | *Vulpes vulpes* | LC | 1 |
| 162 | *Vulpes zerda* | EN | 4 |
| 163 | *Acinonyx jubatus* | CR | 5 |
| 164 | *Caracal caracal* | DD | 1 |
| 165 | *Felis chaus* | LC | 1 |
| 166 | *Felis margarita* | VU | 3 |
| 167 | *Felis silvestris* | LC | 1 |
| 168 | *Panthera pardus* | CR | 5 |
| 169 | *Herpestes ichneumon* | LC | 1 |
| 170 | *Hyaena hyaena* | LC | 1 |
| 171 | *Ictonyx libyca* | EN | 4 |
| 172 | *Mustela subpalmata* | VU | 3 |
| 173 | *Procavia capensis* | LC | 1 |
| 174 | *Equus asinus* | CR | 5 |
| 175 | *Ammotragus lervia* | CR | 5 |
| 176 | *Capra nubiana* | EN | 4 |
| 177 | *Gazella dorcas* | VU | 3 |
| 178 | *Gazella leptoceros* | EN | 4 |

**(B)** Excluded species

**Butterflies**

*Azanus jesous*

*Chilades eleusis*

*Colotis phisadia*

*Elphinstonia charlonia*

*Lycaena thersamon*

*Carcharodus alceae*

*Carcharodus stauderi*

*Colotis danae*

*Pseudotergumia pisidice*

*Anthene amarah*

*Apharitis myrmecophila*

*Calopieris eulimene*

*Charaxes hansali*

*Chazara persephone*

*Colotis chrysonome*

*Colotis liagore*

*Euchloe falloui*

*Gomalia elma*

*Melitaea trivia*

*Pieris brassicae*

*Polyommatus icarus*

*Pseudophilotes abencerragus*

*Pseudophilotes sinaicus*

*Sarangesa phidyle*

*Satyrium jebelia*

*Tomares ballus*

*Zegris eupheme*

*Zizina otis*

**Reptiles**

*Ablepharus rueppellii*

*Atractaspis engaddensis*

*Dasypeltis scabra*

*Dolichophis jugularis*

*Hemidactylus foudaii*

*Hemidactylus mindiae*

*Hemidactylus sinaitus*

*Hemorrohis algirus*

*Hemorrohis nummifer*

*Latastia longicaudata*

*Leptotyphlops macrorhynchus*

*Leptotyphlops nursii*

*Lycophidion capense*

*Mesalina brevirostris*

*Mesalina martini*

*Ophisops elbaensis*

*Ophisops elegans*

*Philochortus zolii*

*Platyceps sinai*

*Psammophis punctulatus*

*Pseuderemias mucronata*

*Pseudocerastes persicus*

*Ptyodactylus ragazzi*

*Rhynchocalamus melanocephalus*

*Telescopus hoogstraali*

*Tropiocolotes nubicus*

*Typhlops vermicularis*

**Mammals**

*Crocidura floweri*

*Crocidura suaveolens*

*Pipistrellus deserti*

*Crocidura religiosa*

*Rhinolophus hipposideros*

*Atelerix algirus*

*Suncus murinus*

*Barbastella leucomelas*

*Nycticeinops schlieffeni*

*Hystrix cristata*

*Hystrix indica*

*Gerbillus nanus*

*Meriones sacramenti*

*Meriones tristrami*

*Vulpes cana*

*Proteles cristata*

*Ictonyx striatus*

*Meles meles*

*Vormela peregusna*

*Genetta genetta*

*Gazella gazella*

**Table S2:** Kendall’s correlation between priority rankings of different surrogate groups (bias-free predictions) using core-area zonation (light grey) and additive-benefit function (dark grey); using Maxent (A) and elastic net (B).

**(A) Maxent**

|  | **Butterflies** | **Reptiles** | **Mammals** | **All species** |
| --- | --- | --- | --- | --- |
| **Butterflies** |  | 0.28 | 0.21 | 0.30 |
| **Reptiles** | 0.47 |  | 0.40 | 0.49 |
| **Mammals** | 0.43 | 0.44 |  | 0.39 |
| **All species** | 0.41 | 0.49 | 0.44 |  |

**(B) elastic net**

|  | **Butterflies** | **Reptiles** | **Mammals** | **All species** |
| --- | --- | --- | --- | --- |
| **Butterflies** |  | 0.31 | 0.20 | 0.31 |
| **Reptiles** | 0.54 |  | 0.40 | 0.54 |
| **Mammals** | 0.38 | 0.48 |  | 0.43 |
| **All species** | 0.47 | 0.56 | 0.43 |  |


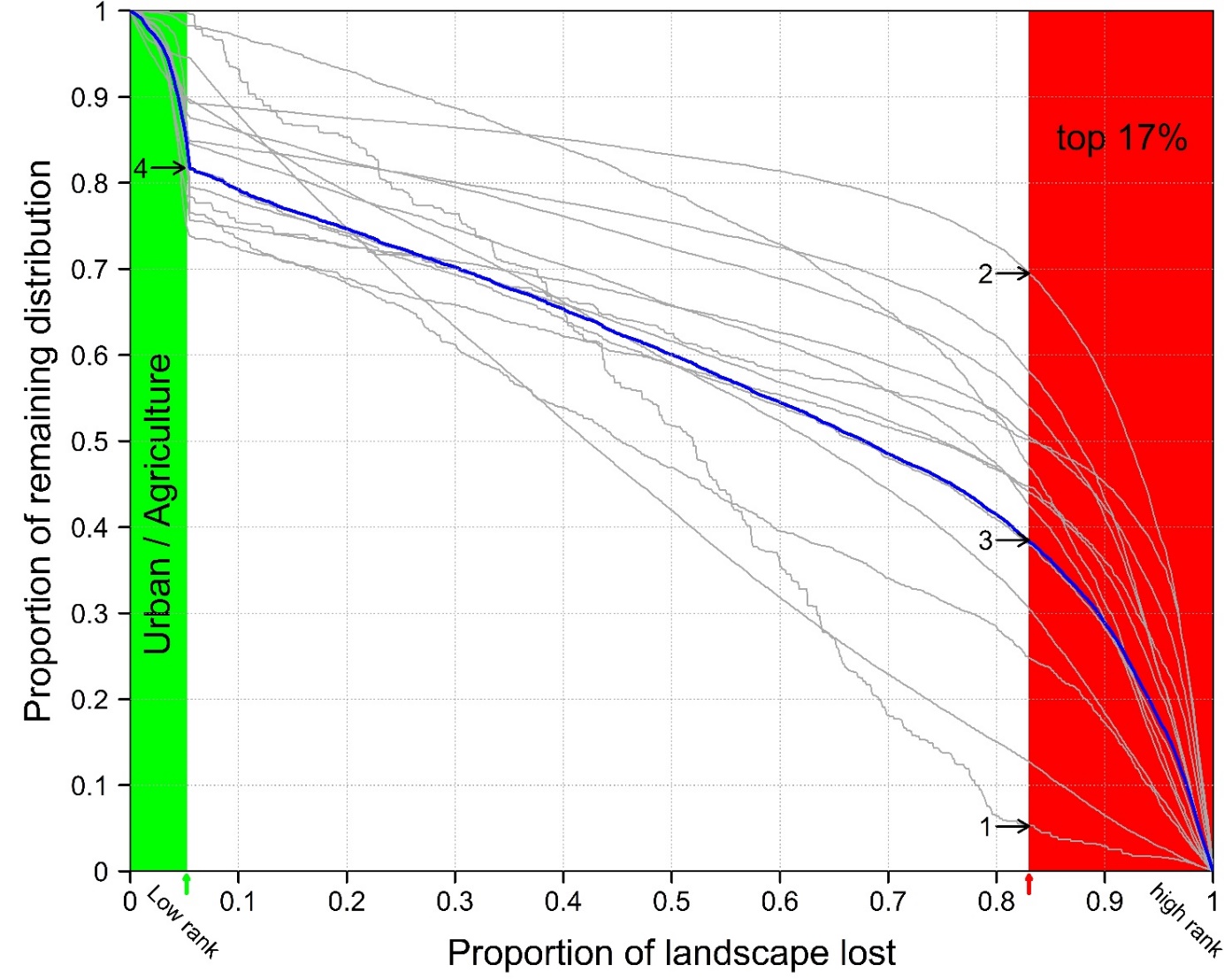


**Figure S1:** An example performance curve produced during the Zonation analysis. This curve quantifies the proportion of features’ original distribution remaining (here, 14 species; y-axis) at each top fraction of the cells (x-axis). The x-axis denotes the priority ranking of the solution, with the highest rank site has a value of one, while the lowest rank site has a value of zero. The per-species performance curve is shown as grey curves, with their overall average in blue. It is possible in some Zonation analyses to handle hundreds of species, and hence the per-species performance curve can be hard to interpret. The overall mean (or per taxonomic group) performance curve is more appropriate in many situations.

It is possible for any percentage of the top (or low) priority fraction of the landscape to get representation level of each species. In this figure, the red area represents the top 17% priority cells. The minimum (1), maximum (2), and the overall mean (3) species representation are shown in the figure. Similarly, the green area represents the urban and agricultural areas in Egypt (Fig. S2.2) which were forced in our analyses to have the lowest priority ranking. The mean species representation in this area is also shown (4). For an example performance curve using the full species list, see Fig. 5.


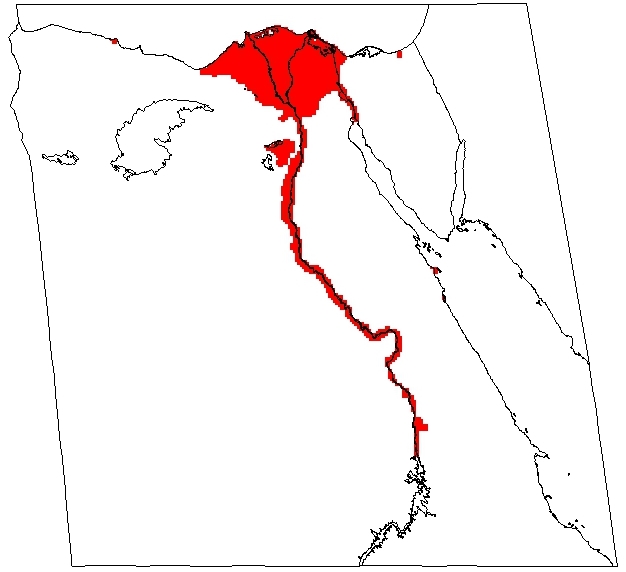


**Figure S2:** Urban and agricultural areas masked during Zonation prioritisation. These areas have high human population density, more polluted, and are of exceptionally high economic value (replacement cost), and hence it is challenging to apply strict conservation actions or construct new PAs in these areas.


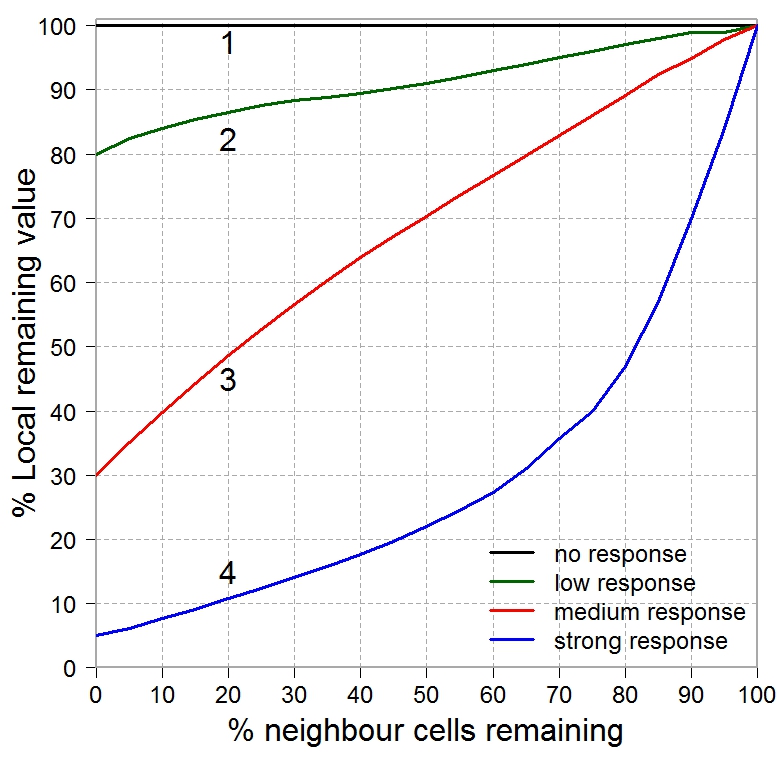


**Figure S3:** Response curves used in the boundary-quality penalty connectivity analyses. These curves describe a range of species sensitivity to habitat loss in neighbour cells, ranging from no response (1) to strong response (4). The x-axis shows the percentage of neighbour cells (specified by three values of radii) remaining: 100 represents no habitat loss; while 0 represents total habitat loss of all neighbour cells. The y-axis shows the percentage reduction in local cell value in response to habitat loss.


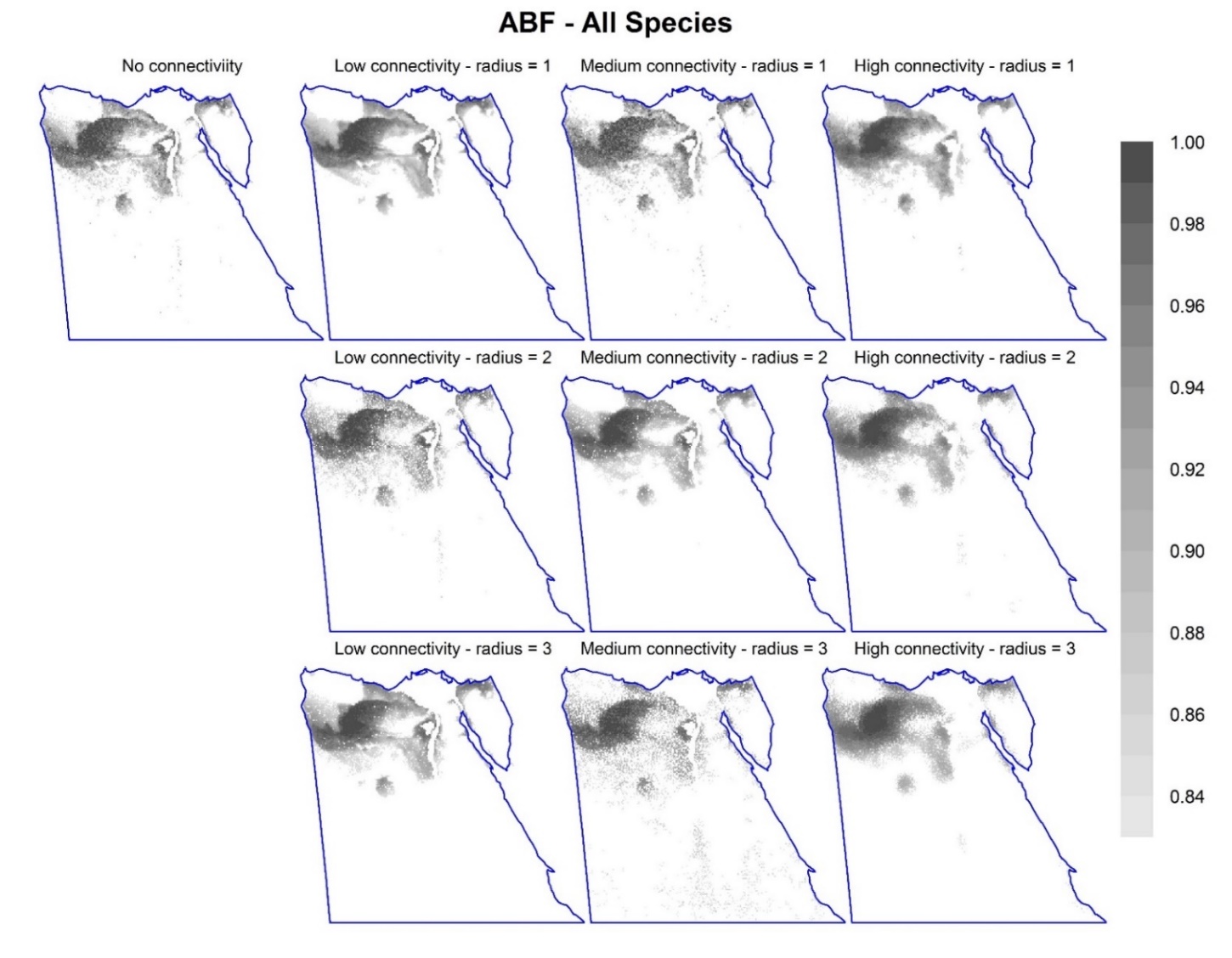


**Figure S4:** The spatial distribution of the top 17% priority cells at different options of connectivity (additive-benefit function; using bias-free predictions of all the study species for Maxent). The top-left map shows the pattern of important sites without connectivity integration. The second to the fourth column is for equivalent maps with steeper response curves (low, medium, and high connectivity; curves 2-4 in Fig. S2.3, respectively); while rows are for different number of effective neighbour cells used (1 to 3). Equivalent maps using core-area zonation are shown in Fig. 2.


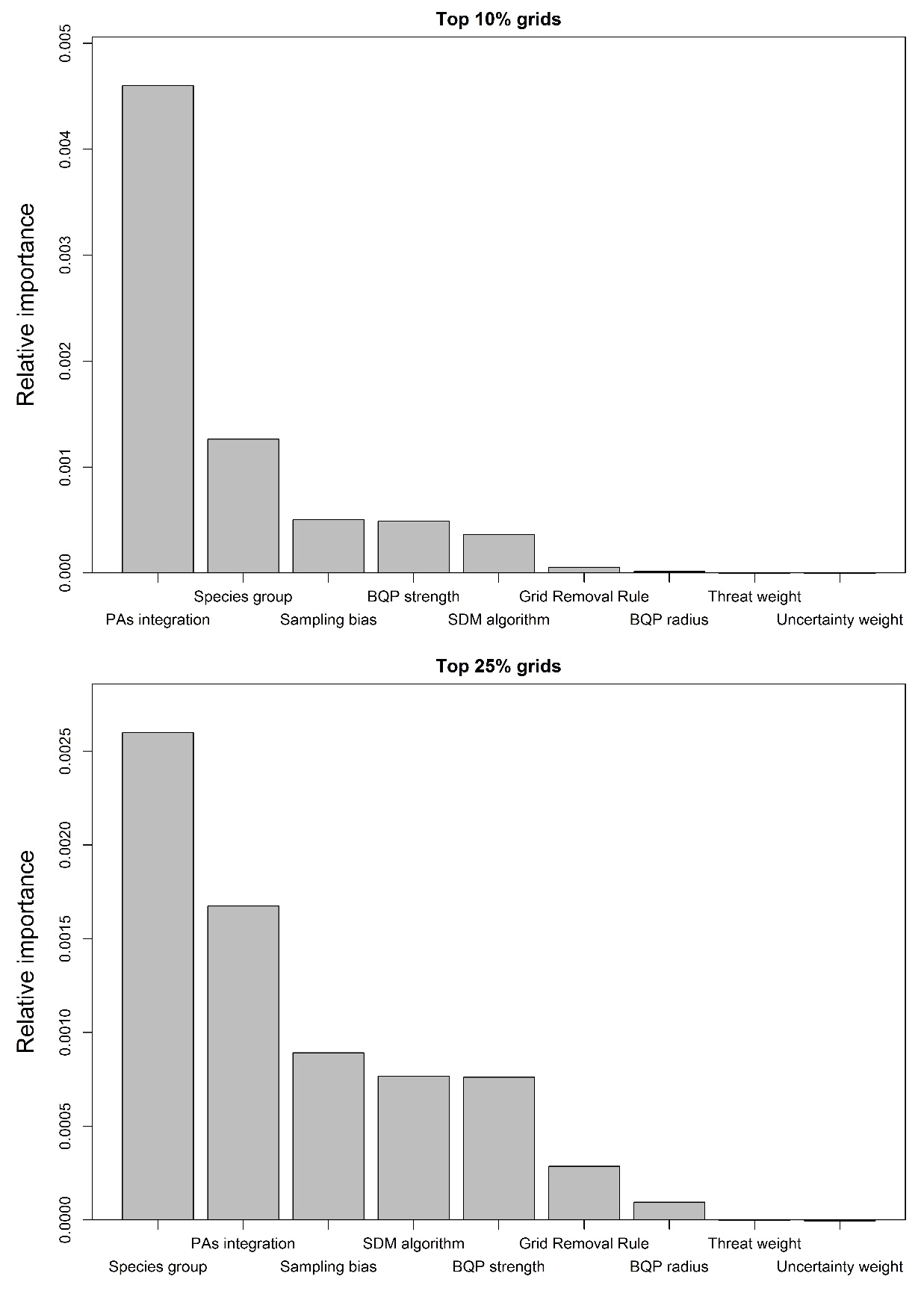


**Figure S5:** Permutation importance of factors affecting the Zonation output’s uncertainty, calculated from a randomForest model (varimp function using party package). Here, the dependent variable is the mean species representation at the top 10% (above) and 25% (below) priority cells.


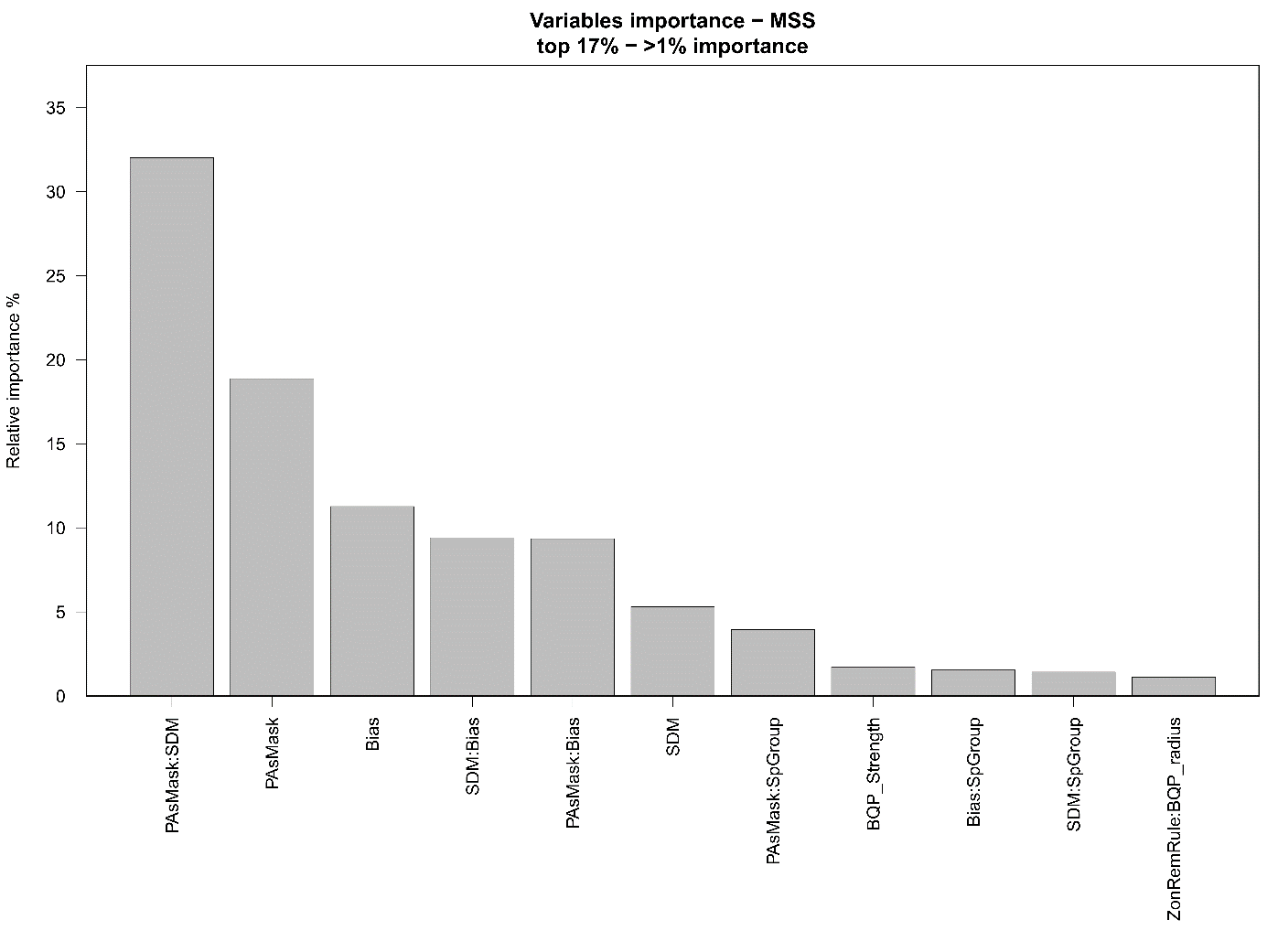


**Figure S6:** The relative importance of various factors affecting the uncertainty of Zonation’s prioritisation (and their first order interactions) using the mean sum of squares from a generalised linear model. Only variables having more than 1% importance are shown.


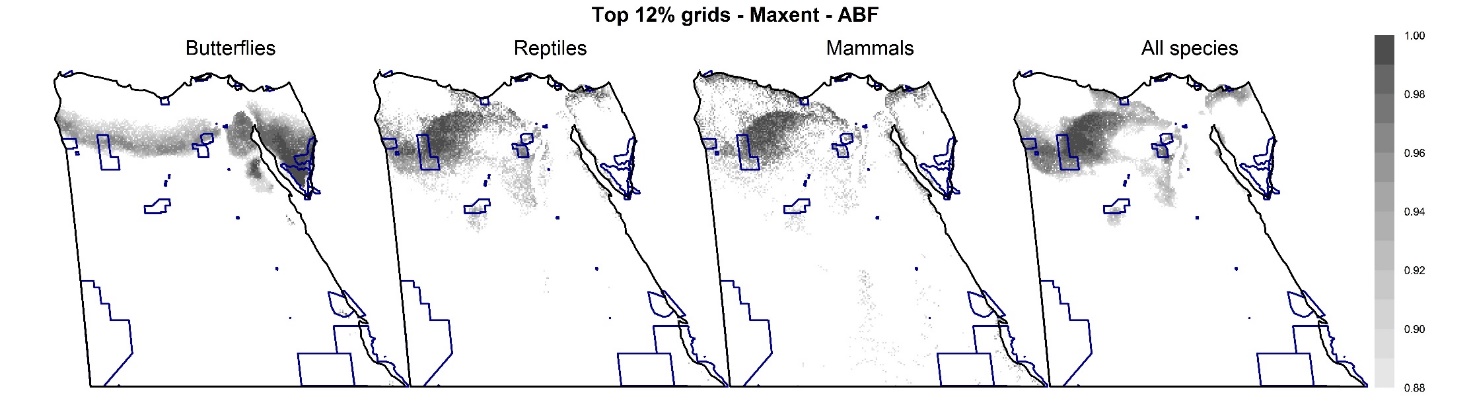


**Figure S7:** The spatial distribution of the top priority cells of equal area to current PAs (the darker, the higher the priority) for different surrogates using additive-benefit function, overlaid with current protected areas in Egypt (blue borders). These maps were prepared using bias-free predictions from Maxent. Equivalent maps using core-area zonation are shown in Fig. 3.


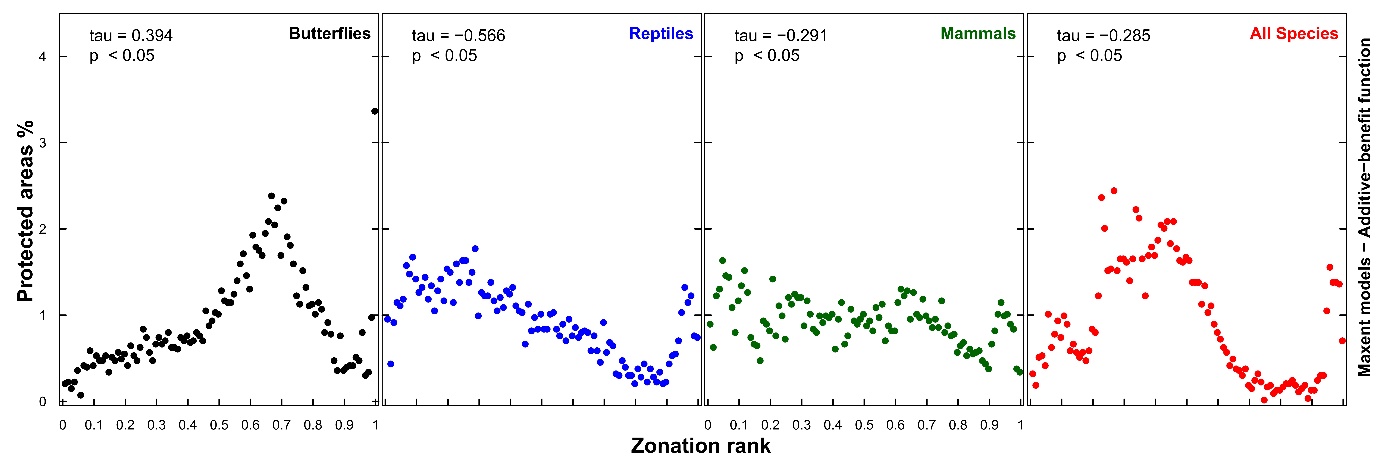


**Figure S8:** The fraction of cells protected at each 1% intervals of Zonation rank for different surrogates using additive-benefit function (using bias-free predictions from Maxent). The number at each panel represents Kendall’s correlation coefficient. Equivalent results for core-area zonation are shown in Fig. 4; while results using elastic net are shown in Fig. S2.9.


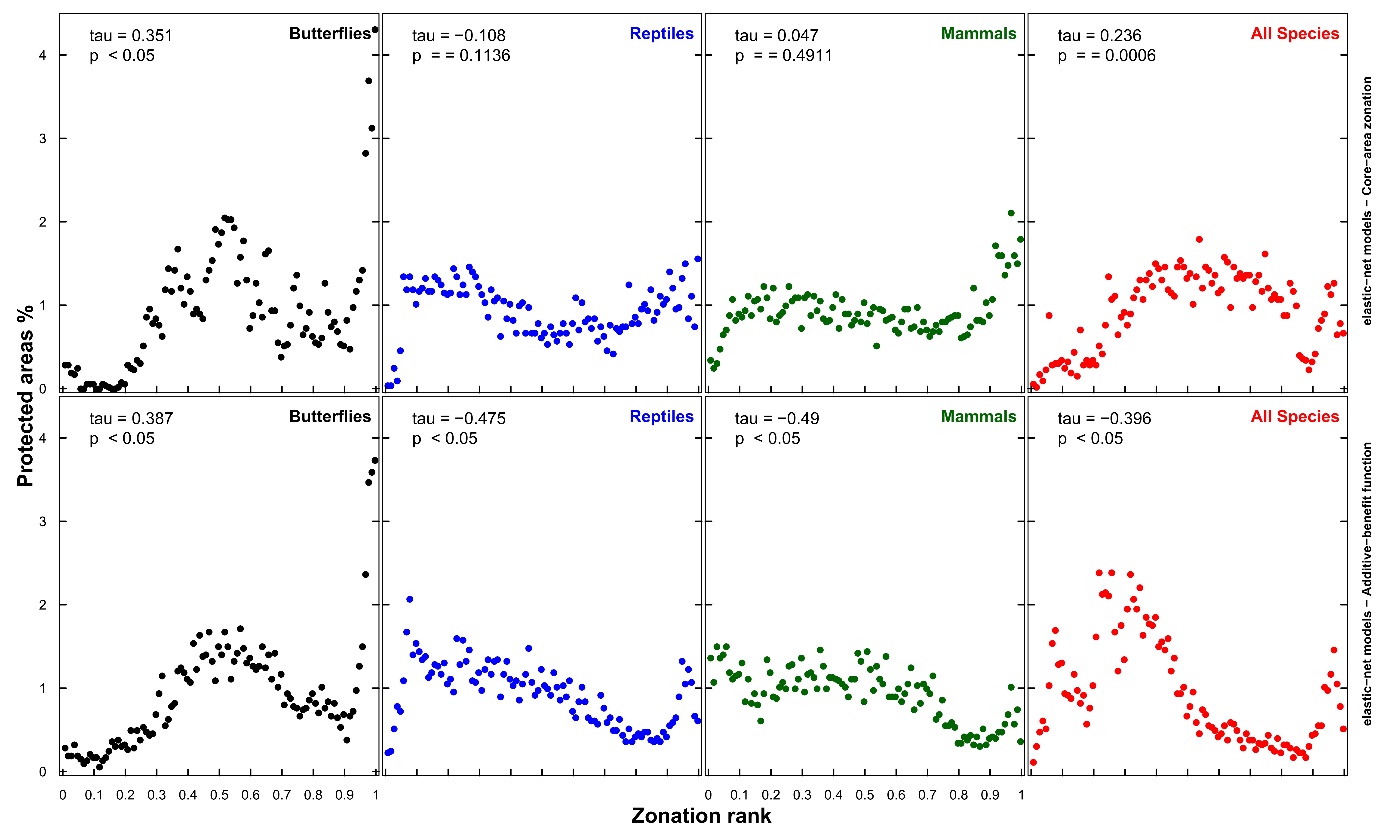


**Figure S9:** The fraction of cells protected at each 1% intervals of Zonation rank for different surrogates using core-area zonation (top row) or additive-benefit function (bottom row), using bias-free predictions for elastic net. The numbers at each panel represent Kendall’s correlation coefficient.


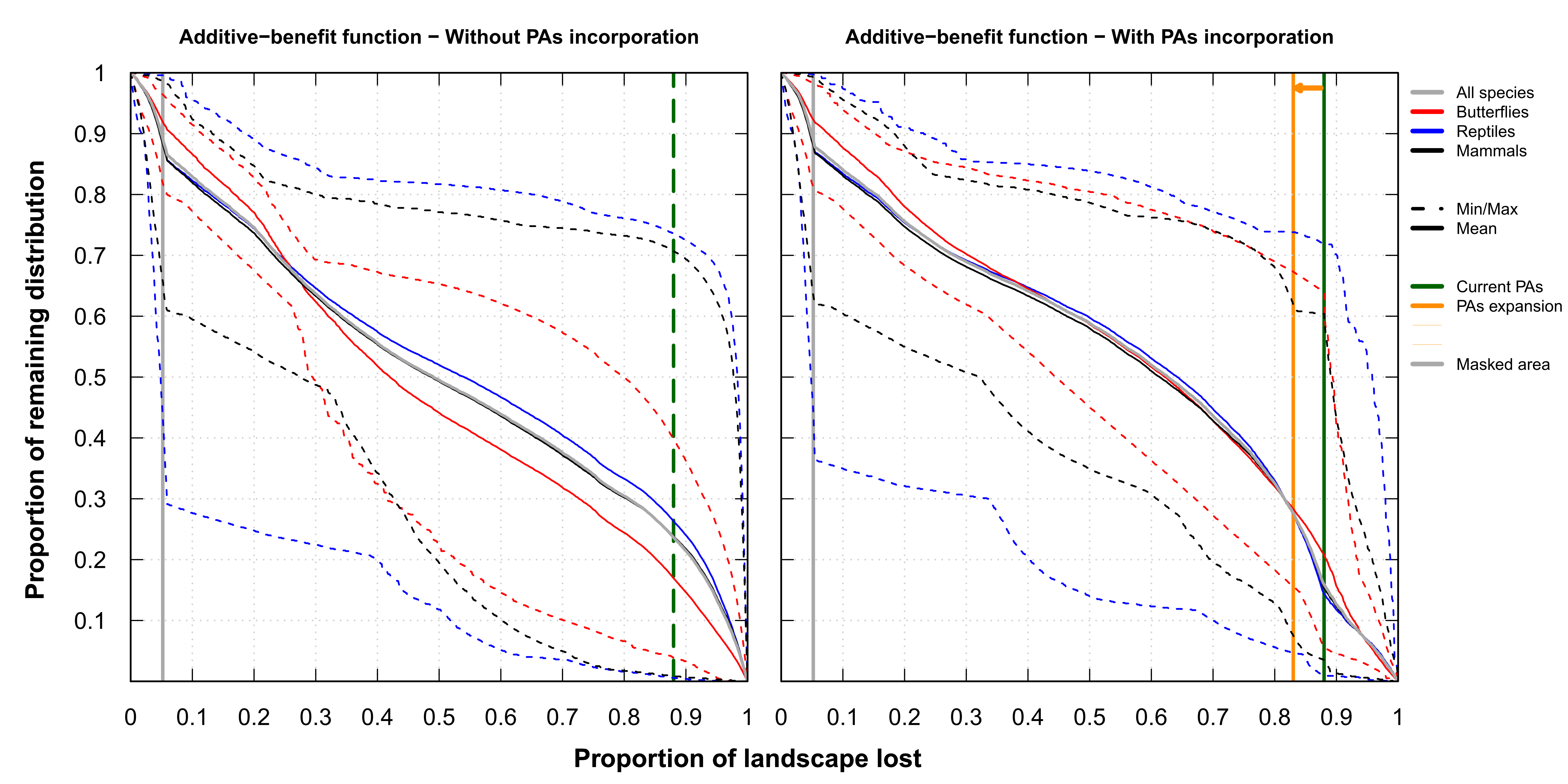


**Figure S10**: Performance curves for Zonation analyses (Maxent – additive-benefit function – all species together). Left panel represents analysis without Egyptian PAs integration. Solid curves represent the average performance curve for all species or per species-group; while dashed lines represent the overall minimum and maximum performance curves per species group. The vertical grey line is for urban and agricultural areas (Fig. S2.2) forced to have low priority value; while the dashed vertical green line represents top priority sites existent in an area equals to the area covered by PAs. The right panel represents equivalent analysis with Egyptian PAs forced to have highest priority scores. The vertical green line represents the area covered by PAs; while the vertical orange line represents the proposed areas for PAs expansion to 17% of Egypt. For equivalent curves using core-area zonation, see Fig. 5. Results for elastic net are shown in Fig. S2.11.


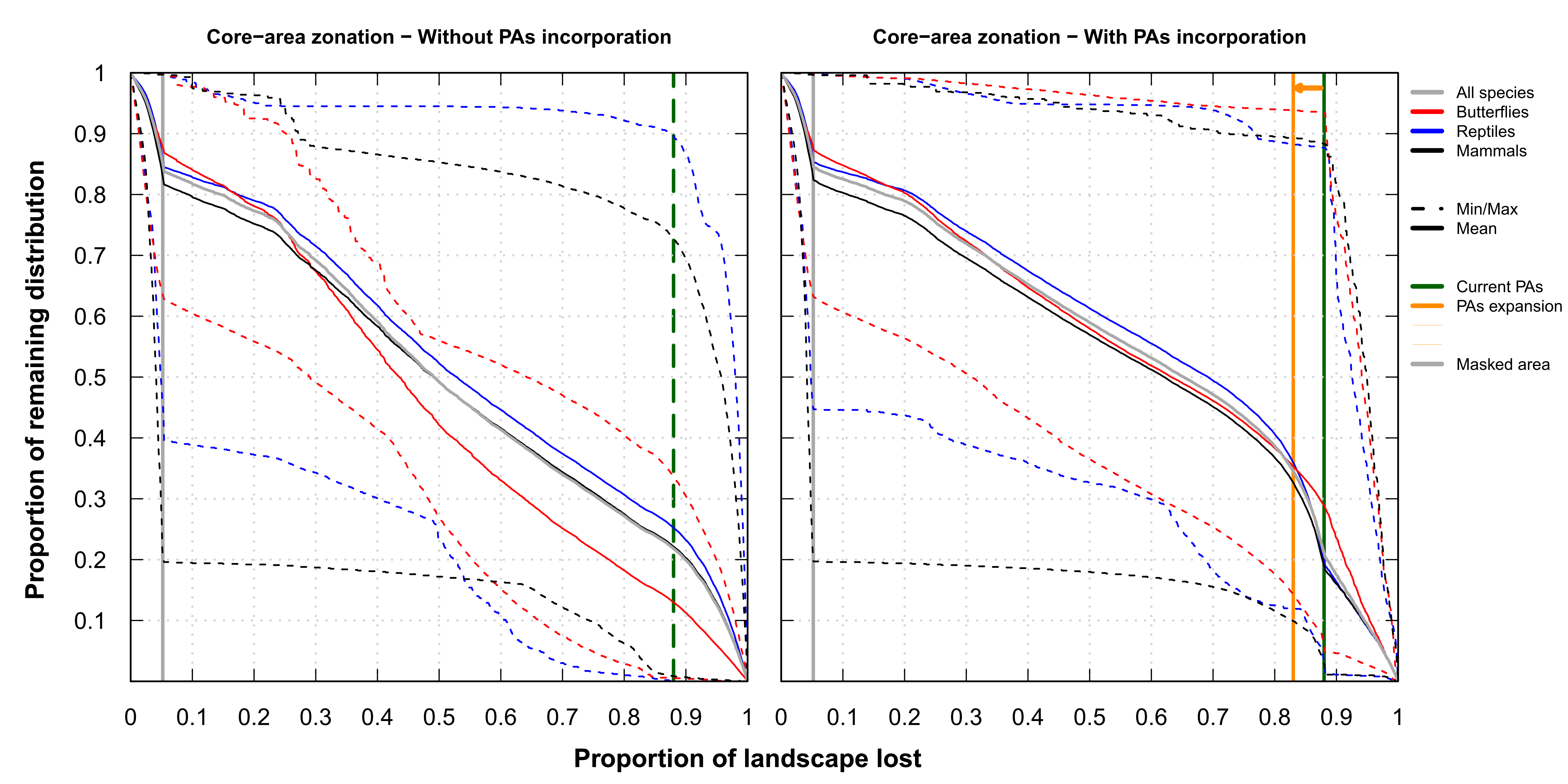


(A)


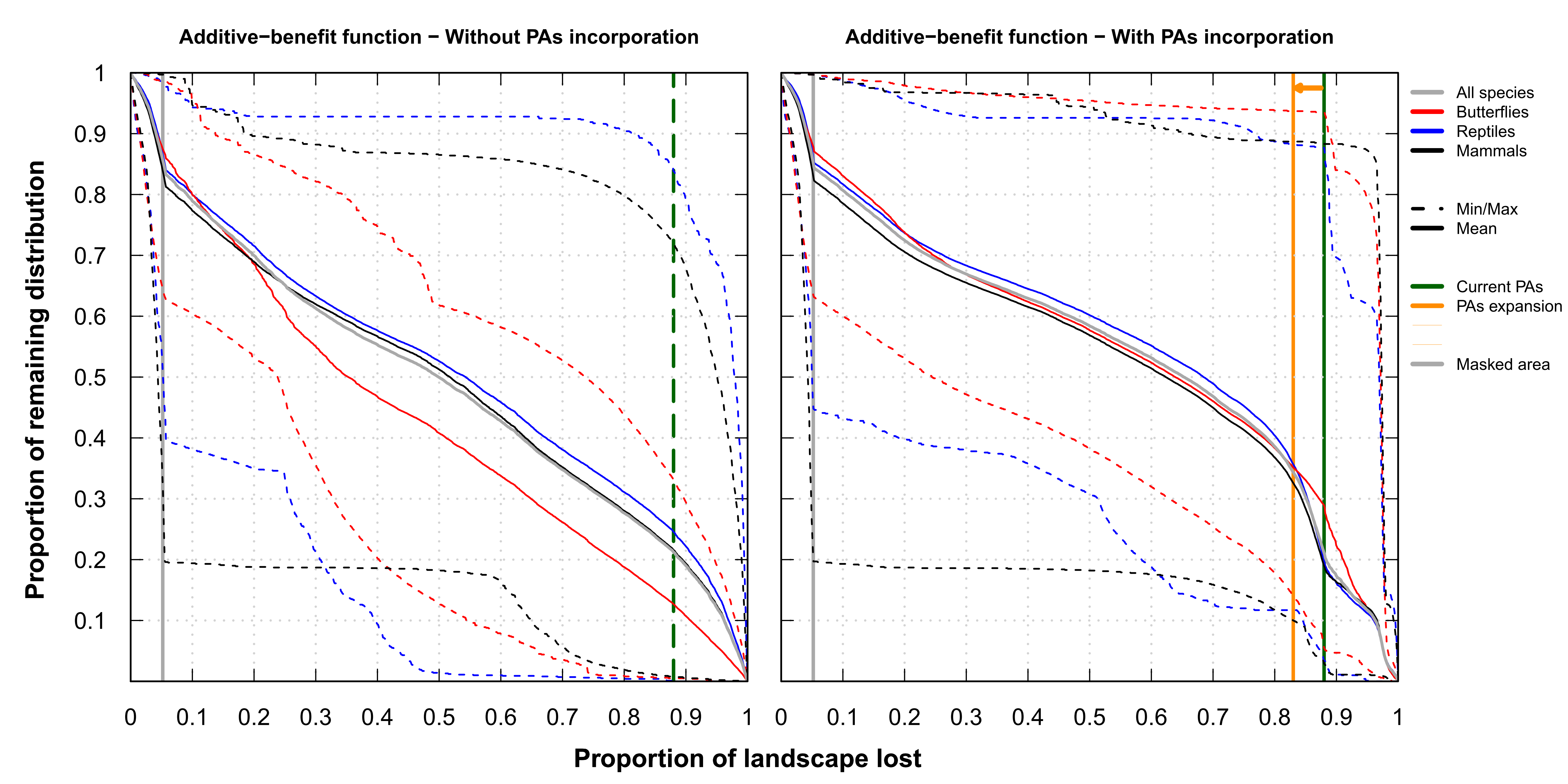


(B)

**Figure S11:** Performance curves for elastic-net models using core-area zonation (A) and additive benefit function (B). For more details, see Fig. 5 and main text.


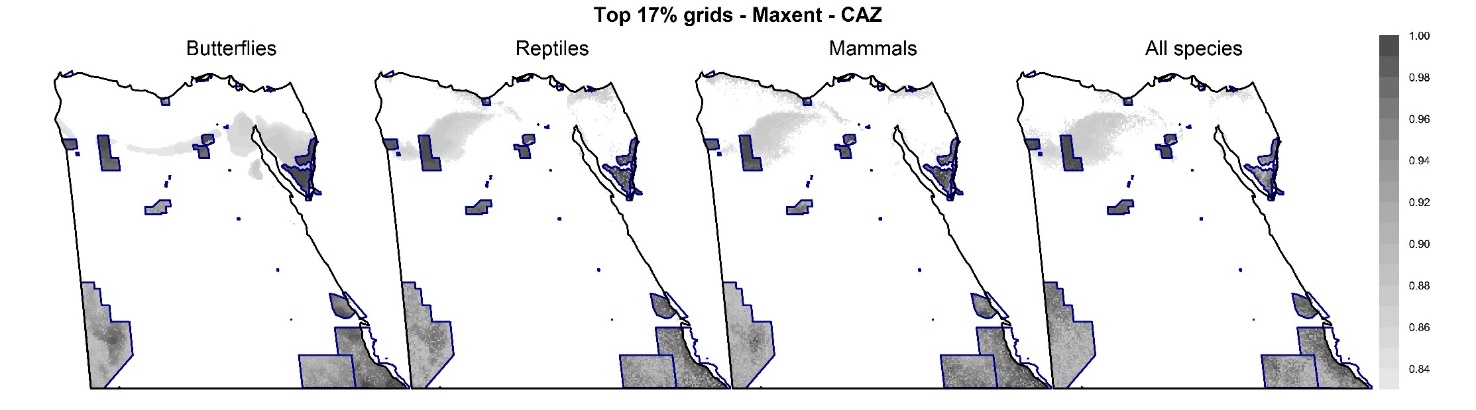


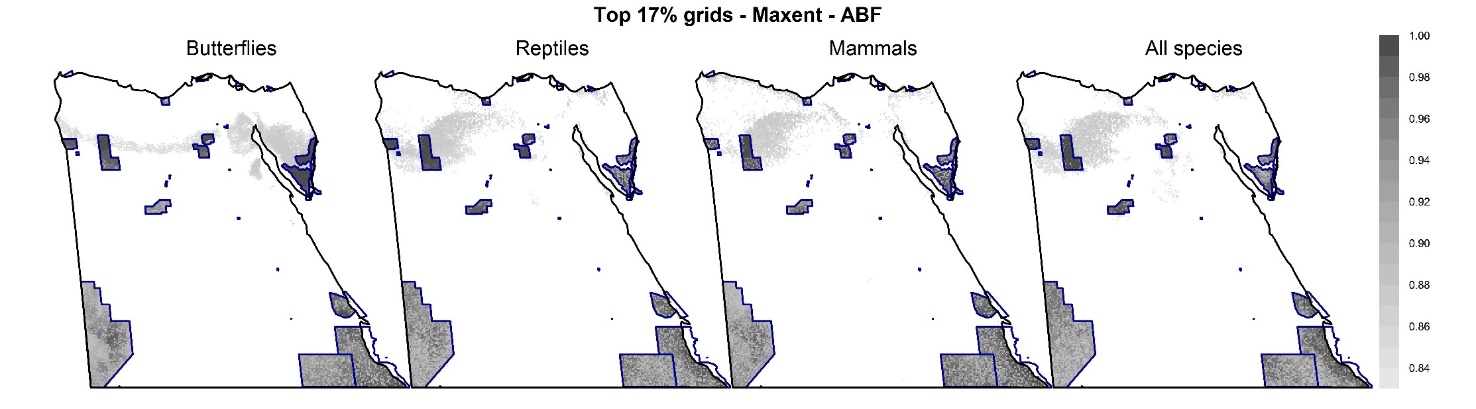


**Figure S12:** Potential areas for Egyptian PAs expansion (to 17% of Egypt) using core-area zonation (above) and additive-benefit function (below) for each surrogate. All maps use bias-free predictions from Maxent. Egyptian PAs (shown in blue) were always forced to have the highest ranking. Grey areas outside PAs represent potential areas for Egyptian PAs expansion. The ranking of these maps are added together to summarise the overall pattern of important sites outside PAs (see Fig. 6, and similarly Fig. S2.13 for elastic net).

**
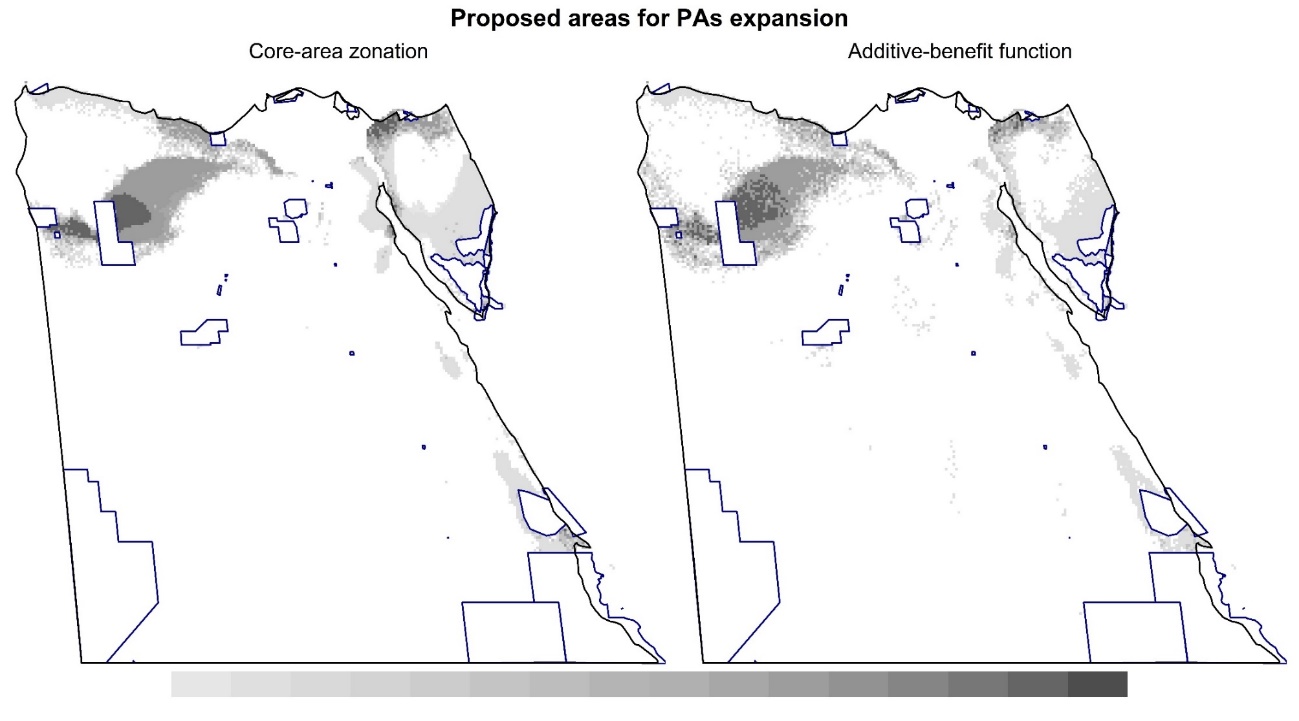
**

**Figure S13:** The overall pattern of top priority sites using core-area zonation (left) or additive-benefit function (right), both using bias-free predictions from elastic net. Each map shows the summed rankings of the top 17% sites from the four surrogates used. Top priority sites within current protected areas (blue borders) are not shown to highlight the overall pattern of potential areas for PAs expansion (the darker the colour, the higher the cumulative rank of the site using the four surrogates). For equivalent maps using Maxent, see Fig. 6.


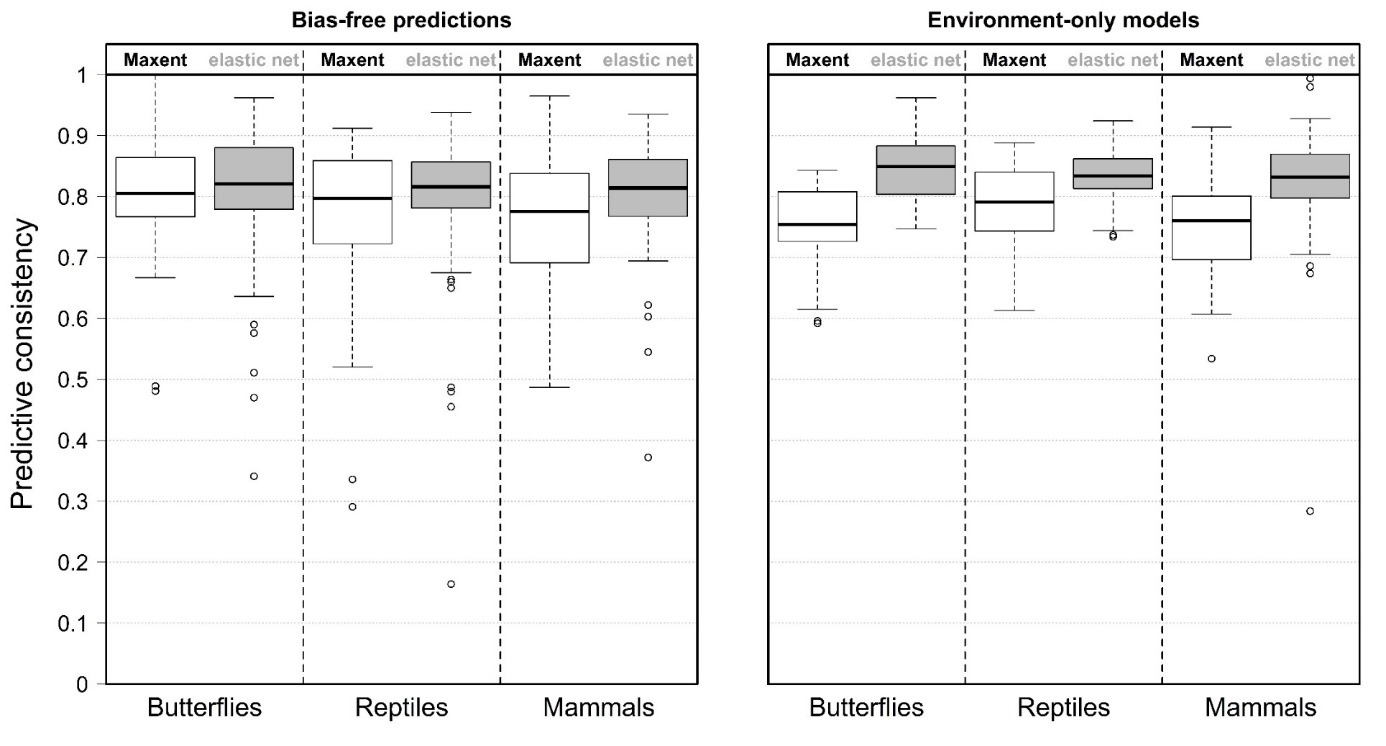


**Figure S14:** Boxplots for the predictive consistency of cross-validated models with (left) and without (right) correction for sampling bias. Predictions from Maxent are shown in white boxes, while elastic-net is shown in grey boxes. The median predictive consistency is about 0.8, with relatively higher median consistency for elastic net (environment-only models). Predictive consistency was one method of weighting species in Zonation, see the main text for details.

**
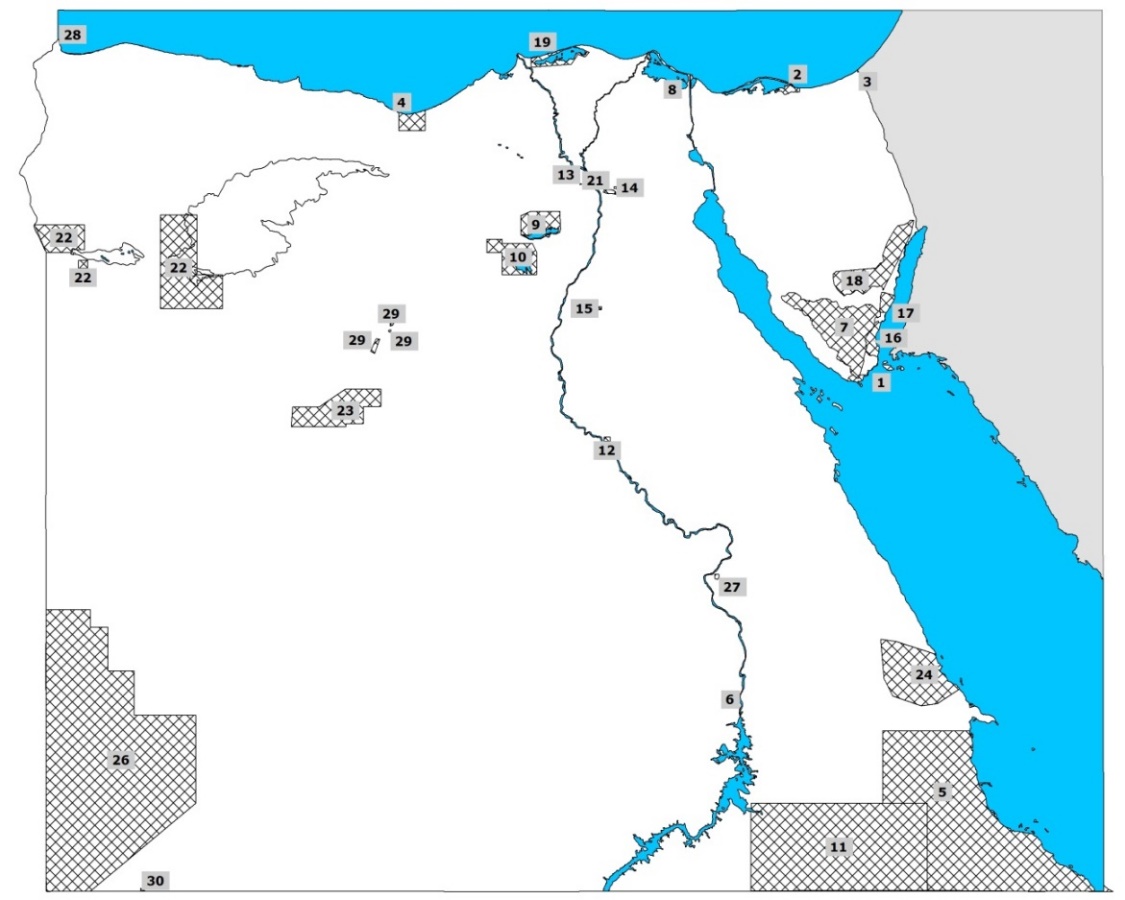
**

| **No.** | **Protectorate Name** | **Declaration Date** | **Area Km²** | **Governorate** |
| --- | --- | --- | --- | --- |
| 1 | Ras Mohamed National Park | 1983 | 850 | South Sinai |
| 2 | Zaranik Protectorate | 1985 | 230 | North Sinai |
| 3 | Ahrash Protectorate | 1985 | 8 | North Sinai |
| 4 | El-Omayed Protectorate | 1986 | 700 | Matrouh |
| 5 | Elba National Park | 1986 | 35600 | Red Sea |
| 6 | Saluga and Ghazal Protectorate | 1986 | 0.5 | Aswan |
| 7 | St. Katherine National Park | 1988 | 4250 | South Sinai |
| 8 | Ashtum El-Gamil Protectorate | 1988 | 180 | Port Said |
| 9 | Lake Qarun Protectorate | 1989 | 250 | El Fayoum |
| 10 | Wadi El-Rayan Protectorate | 1989 | 1225 | El Fayoum |
| 11 | Wadi Allaqi Protectorate | 1989 | 30000 | Aswan |
| 12 | Wadi El-Assuti Protectorate | 1989 | 35 | Assuit |
| 13 | El Hassana Dome Protectorate | 1989 | 1 | Giza |
| 14 | Petrified Forest Protectorate | 1989 | 7 | Cairo |
| 15 | Sannur Cave Protectorate | 1992 | 12 | Beni Suef |
| 16 | Nabq Protectorate | 1992 | 600 | South Sinai |
| 17 | Abu Galum Protectorate | 1992 | 500 | South Sinai |
| 18 | Taba Protectorate | 1998 | 3595 | South Sinai |
| 19 | Lake Burullus Protectorate | 1998 | 460 | Kafr El Sheikh |
| 20 | Nile Islands Protectorates | 1998 | 160 | All Governorates on the Nile |
| 21 | Wadi Degla Protectorate | 1999 | 60 | Cairo |
| 22 | Siwa | 2002 | 7800 | Matrouh |
| 23 | White Desert | 2002 | 3010 | Matrouh |
| 24 | Wadi El-Gemal/Hamata | 2003 | 7450 | Red Sea |
| 25 | Red Sea Northern Islands | 2006 | 1991 | Red Sea |
| 26 | El-Gilf El-Kebir | 2007 | 48523 | New Valley |
| 27 | El-Dababya | 2007 | 1 | Qena |
| 28 | El-Salum Gulf | 2010 | 383 | Matrouh |
| 29 | El-Wahat El-Bahreya | 2010 | 109 | 6th October |
| 30 | Mount Kamel Meteor Protectorate | 2012 | 1 | New Valley |

**Figure S15**: Current Egyptian protected areas.


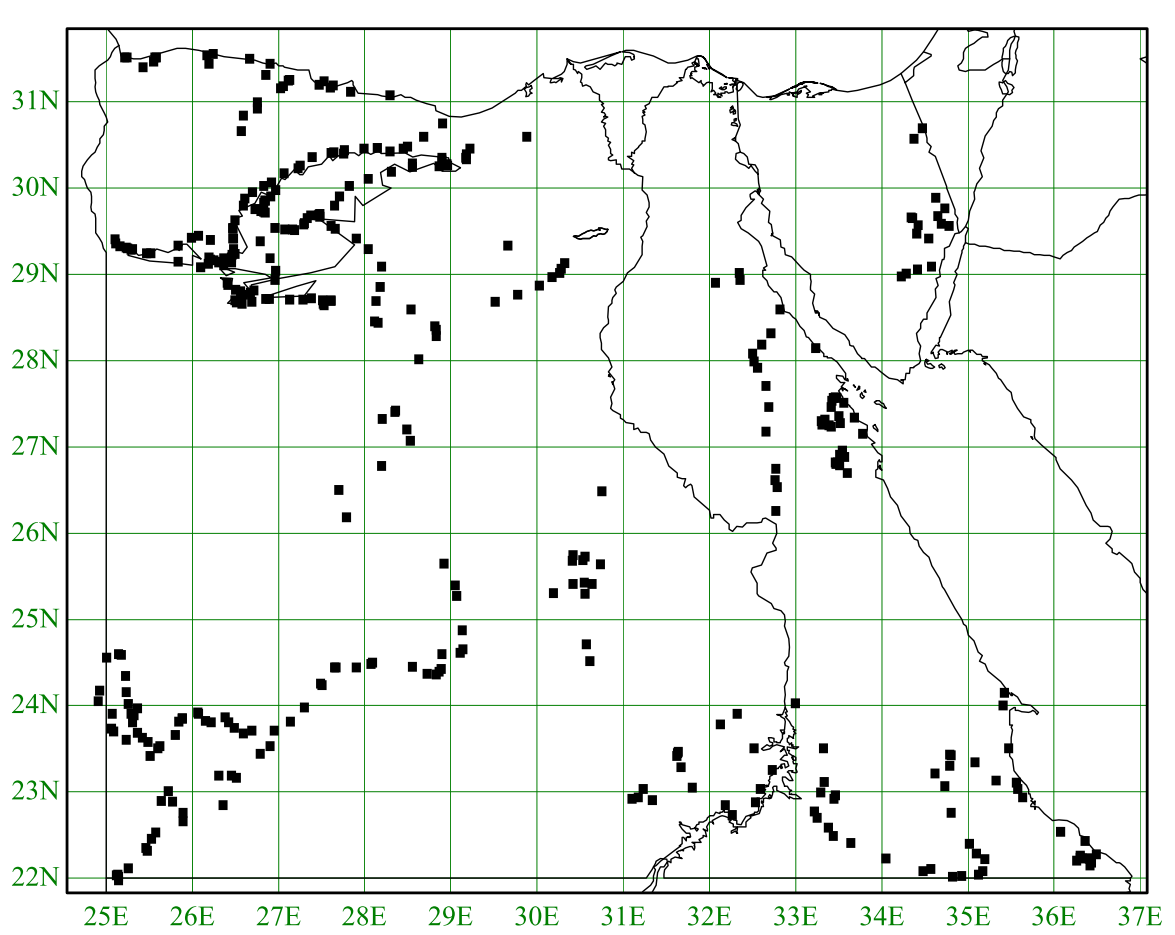


**Figure S16:** Field evaluation sites visited during protected areas identification mission between December 1996 and April 1998. (Sherif Baha El Din, pers. comm.).


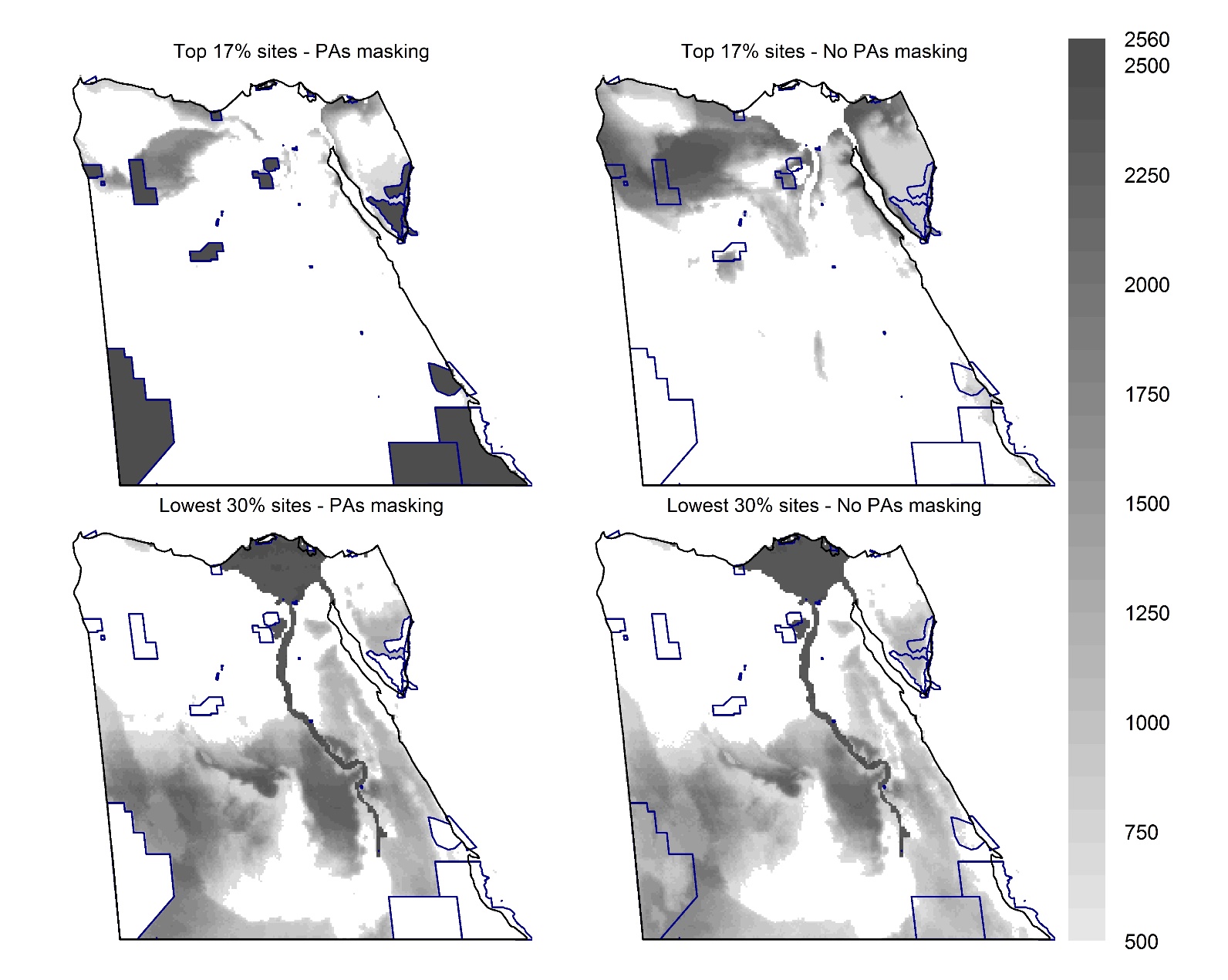


**Figure S17:** The frequency with which each cell was located at the top or lowest priority sites across all prioritization analyses performed. For each of the 2,560 Zonation analysis, the top 17% (above) or lowest 30% (below) priority sites were determined and the number of times each cell was chosen is reported, either with forcing PAs to have highest scores (left) or not (right). Only cells chosen in more than 500 zonation solutions are shown.

**Appendix S1:** Data sources

List of literature resources used for extracting Egyptian records.

| ACR (2015) **African Chiroptera Report**. 2015. AfricanBats, Pretoria, i - xix, 1 - 7001 pp.  Alfieri A. (1942) **Quatre lépidoptères nouveaux pour la faune du Sinai**. Bulletin de la Société Fouad 1er Entomologie, 26: 163-166.  Al-Hussaini A.H. (1959) **The vertebrate fauna of the Bahariya Oasis (excluding fishes and mammals).** Ain Shams Science Bull., 4:103-107.  Ali R.A. (2011) **Molecular Phylogenetic Relationship Between and Within the Fruit Bat (*Rousettus aegyptiacus*) and the Lesser Tailed Bat (*Rhinopoma hardwickei*) Deduced From RAPD-PCR Analysis**. Journal of American Science, 7(10):678-687.  Andersen K. (1906) **On some new or little-known bats of the genus *Rhinolophus* in the collection of the Museo Civico, Genoa**. Annali del Musei Civico di Storia Naturale di Genova, (3a): 173-195.  Anderson J. (1902) **Zoology of Egypt: Mammalia (compiled by W.E. de Winton)**. London: Hugh Rees Ltd.  Andres A. & Seitz A. (1925) **Die Lepidopteren-Fauna Aegyptens.** Nachtrag zum ersten Teil. Senckenbergiana, 7(1/2): 54-61.  Andres A. (1912) **Verzeichnis der bis jetzt in Aegypten beobachten Schmetterlinge.** Bulletin de la Société entomologiques d'Egypte, 5: 53-114.  Awadallah A.M., Azab A.K. & El Nahal A.K.M. (1970) **Studies on the pomegranate butterfly, *Virachola livia* (Klug)**. Bulletin de la Société entomologiques d'Egypte, 54: 545-567.  Badir M. A. & EL-Nagar M. H. (1994) **Some ecological studies on the fauna of Saint Kathrine area in South Sinai with special reference to the Agamid lizard *Agama stellio***. J. Egypt Ger. Soc. Zool., 13 (A), 67:84.  Baha El Din S. (2006) **A guide to the reptiles and amphibians of Egypt**. The American University in Cairo Press, Cairo.  Baha El Din S.M. (1999) **A new species of *Tropiocolotes* (Reptilia: Gekkonidae) from Egypt**. Zoology in the Middle East, 19: 17-26.  Baha El Din S.M. (2001) **A synopsis of African and south Arabian geckos of the genus *Tropiocolotes* (Reptilia: Gekkonidae), with a description of a new species from Egypt**. Zoology in the Middle East, 22: 45-56.  Baha El Din S.M. (2003) **A new species of *Hemidactylus* (Squamata: Gekkonidae) from Egypt**. African Journal of Herpetology, 52: 39-47.  Baha El Din S.M. (2005) **An overview of Egyptian species of *Hemidactylus* (Gekkonidae), with the description of a new species from the high mountains of South Sinai**. Zoology in the Middle East, 34: 11-26.  Baha El Din S.M. (2011) **Distribution and recent range extension of *Natrix tessellata* in Egypt**. Mertensiella, 18: 401-403.  Baharav D. & Meiboom U. (1981) **The status of the Nubian ibex *Capra ibex nubiana* in the Sinai desert**. Biological Conservation, 20(2): 91-97.  Bajer A., Harris P.D., Behnke J.M., et al. (2006) **Local variation of haemoparasites and arthropod vectors, and intestinal protozoans in spiny mice (*Acomys dimidiatus*) from four montane wadis in the St Katherine Protectorate, Sinai, Egypt**. Journal of Zoology, 270: 9-24.  Barnard C.J., Sayed E., et al. (2003) **Local variation in helminth burdens of Egyptian spiny mice (*Acomys cahirinus dimidiatus*) from ecologically similar sites: relationships with hormone concentrations and social behaviour**. Journal of Helminthology, 77(3):197-207.  Basuony M.I. & Saleh M.A. (2005) **Biodiversity of the southern sector of the Egyptian Eastern Desert.** Egyptian Journal of Zoology, 44: 99-123.  Basuony M.I. (2003) **Ecological distribution and zoogeographical relationships of mammalian assemblages in two wetland areas in northern Egypt**. Egyptian Journal of Zoology, 40: 575-599.  Basuony M.I. (2004) **New distributional records of the shrews *Crocidura nana* Dobson, 1890 and *Suncus murinus* (Linnaeus, 1766) from Egypt**. Al-Azhar Bulletin of Science, 15(2): 11-17.  Basuony M.I. (2013) **Food and feeding ecology of the Egyptian Mongoose, *Herpestes ichneumon* (Linnaeus, 1758) in Egypt**. Journal of Applied Sciences Research, 9(11): 5811-5816.  Basuony M.I., Gilbert F. & Zalat S. (2010). **Mammals of Egypt: atlas, red data listing & conservation**. BioMAP & CultNat, EEAA & Bibliotheca Alexandrina, Cairo. 286 pp.  Bedir M.A., El-Naggar M.H., Kayed A.N. & Orabi G.M. (1999) **Ecological studies on two lizard species inhabiting Wadi El-Arbein at Saint Katherine Area, South Sinai with special reference to biotic elements**. J. Egypt. Ger. Soc. Zool., 28(A): 485-503.  Benda P., Abi-Said M., Bartonička T., Bilgin R., Faizolahi K., Lučan R.K., Nicolaou H., Reiter A., Shohdi W.M., Uhrin M., Horáček I. (2011) ***Rousettus aegyptiacus* (Pteropodidae) in the Palaearctic: list of records and revision of the distribution range**. Vespertilio, 15: 3–36.  Benda P., Al-Juaid M.M., Reiter A., Nasher A.K. (2011) **Noteworthy records of bats from Yemen with description of a new species from Socotra**. Hystrix, 22(1):23–56.  Benda P., Andreas M., Kock D., Lučan R., Munclinger P., Nová P., Obuch J., Ochman K., Reiter A., Uhrin M., Weinfurtová D. (2006) **Bats (Mammalia: Chiroptera) of the Eastern Mediterranean. Part 4. Bat fauna of Syria: distribution, systematics, ecology**. Acta Societatis Zoologica Bohemicae, 70:1–329.  Benda P., Andriollo T., Ruedi M. (2014) **Systematic position and taxonomy of *Pipistrellus deserti* (Chiroptera: Vespertilionidae)**. Mammalia, 79(4):419-438.  Benda P., Červený J., Konečný A., Reiter A., Ševčík M., Uhrin M., Vallo P. (2010) **Some new records of bats from Morocco (Chiroptera)**. Lynx, 41:151-166.  Benda P., Dietz C., Andreas M., Hotový J., Lučan R., Maltby A., Meakin K., Truscott J., Vallo P. (2008) **Bats (Mammalia: Chiroptera) of the Eastern Mediterranean and Middle East. Part 6. Bats of Sinai (Egypt) with some taxonomic, ecological and echolocation data on that fauna**. Acta Societatis Zoologicae Bohemicae, 72(1-2):1-103.  Benda P., Faizolâhi K., Andreas M., Obuch J., Reiter A., Ševčík M., Uhrin M., Vallo P., Ashrafi S. (2012) **Bats (Mammalia: Chiroptera) of the Eastern Mediterranean and Middle East. Part 10. Bat fauna of Iran**. Acta Societatis Zoologicae Bohemicae, 76:163–582.  Benda P., Gvoždík V. (2010) **Taxonomy of the genus *Otonycteris* (Chiroptera: Vespertilionidae: Plecotini) as inferred from morphological and mtDNA data**. Acta Chiropterologica, 12(1):83-102.  Benda P., Hanák V., Andreas M., Reiter A., Uhrin M. (2004) **Two new species of bats (Chiroptera) for the fauna of Libya: *Rhinopoma hardwickii* and *Pipistrellus rueppellii***. Myotis, 41–42: 109-124.  Benda P., Hanák V., Červený J. (2011) **Bats (Mammalia: Chiroptera) of the Eastern Mediterranean and Middle East. Part 9. Bats from Transcaucasia and West Turkestan in collection of the National Museum, Prague**. Acta Societatis Zoologicae Bohemicae, 75:159–222.  Benda P., Kiefer A., Hanák V., Veith M. (2004) **Systematic status of African populations of long-eared bats, genus *Plecotus* (Mammalia: Chiroptera)**. Folia Zoologica, 53(1):1–48.  Benda P., Lučan R. K., Shohdi W. M., Porteš M., Horáček I. (2014) **Microbats of the Western Oases of Egypt, Libyan Desert**. Vespertilio, 17:45–58.  Benda P., Lučan R.K., Obuch J., Reiter A., Andreas M., Bačkor P., Bohnenstengel T., Eid E.K., Ševčík M., Vallo P., et al. (2010) **Bats (Mammalia: Chiroptera) of the Eastern Mediterranean and Middle East. Part 8. Bats of Jordan: fauna, ecology, echolocation, ectoparasites**. Acta Societas Zoologicae Bohemicae, 74:185–353.  Benda P., Reiter A., Al-Jumaily M., Nasher A.-K., Hulva P. (2009) **A new species of mouse-tailed bat (Chiroptera: Rhinopomatidae: *Rhinopoma*) from Yemen**. Journal of the National Museum (Prague), Natural History Series, 177 (6):53–68.  Benda P., Ruedi M. (2004) **New data on the distribution of bats (Chiroptera) in Morocco**. Lynx, 35:13-44.  Benda P., Spitzenberger F., Hanák V., Andreas M., Reiter A., Ševčík M., Šmíd J., Uhrin M. (2014) **Bats (Mammalia: Chiroptera) of the Eastern Mediterranean and Middle East. Part 11. On the bat fauna of Libya II**. Acta Societatis Zoologicae Bohemicae, 78:1–162.  Benda P., Vallo P. (2012) **New look on the geographical variation in *Rhinolophus clivosus* with description of a new horseshoe bat species from Cyrenaica, Libya**. Vespertilio, 16: 69-96.  Benda P., Vallo P., Hulva P., Horáček I. (2012) **The Egyptian fruit bat *Rousettus aegyptiacus* (Chiroptera: Pteropodidae) in the Palaearctic: geographical variation and taxonomic status**. Biologia, 67(6):1230–1244.  Benda P., Vallo P., Reiter A. (2011) **Taxonomic revision of the genus *Asellia* (Chiroptera: Hipposideridae) with a description of a new species from Southern Arabia**. Acta Chiropterol, 13(2):245–270.  Benyamini D. (1984) **The butterflies of the Sinai Peninsula (Lep. Rhopalocera).** Nota Lepidoptera, 7(4): 309-321.  Benyamini D. (1999) **The biology and conservation of *Iolana alfierii* Wiltshire, 1948: the Burning Bush Blue (Lepidoptera: Lycaenidae).** Linneana Belgica, 17(4): 119-134.  Bergmans W. (1994) **Taxonomy and biogeography of African fruit bats (Mammalia, Megachiroptera). 4. The genus *Rousettus* Gray, 1821**. Beaufortia, 44:79-126.  Bonhote J.L. (1909) **On a small collection of mammals from Egypt**. Proceedings of the Zoological Society of London, 79(4)788-802.  Boyd A.W. (1917) **Six month’s collecting between Ismailia and El Arish**. Bulletin de la Société entomologiques d'Egypte, 5: 98-119.  Churcher C.S. (1991) **The Egyptian fruit bat *Rousettus aegyptiacus* in Dakhleh Oasis, Western Desert of Egypt**. Mammalia, 551:139-143.  DeBlase A. (1972) ***Rhinolophus euryale* and *R. mehelyi* (Chiroptera: Rhinolophidae) in Egypt and Southwest Asia**. Israel Journal of Zoology, 21(1):1-12.  Debski B. (1919) **Quelques observations sur *Teracolus fausta*, *Melitaea didyma* et *Azanus ubaldus*.** Bulletin de la Société entomologique de France, 1919: 33-43.  Demaison L. (1895) **Note sur les Lépidoptères d’Egypte.** Bulletin de la Société entomologique de France, 1895: 51-62.  Dietz C. (2005) **Bats Final Report - Operation Wallacea. Sinai 2005**. 23 pp.  Dornburg A., Colosi J.G., Maser C., Reesel A.T., Watkins-Colwell G.J. (2011) **A survey of the Yale Peabody Museum collection of Egyptian mammals collected during construction of the Aswan high dam, with an emphasis on material from the 1962–1965 Yale University prehistoric expedition to Nubia**. Bulletin of the Peabody Museum of Natural History, 52(2):255-272.  El-Gabbas A. & Gilbert F. (2016) **The Desert Beauty *Calopieris eulimene*: a butterfly new to Egypt (Insecta: Lepidoptera)**. Zoology in the Middle East, 62(3): 279–281.  Farid M. (1977) **On some fishes, Amphibia, and reptilia from Siwa Oasis**. Proc. Zool. Soc. A.R.E, 6: 234-231.  Fathy W. (2006) **Studies of the genus *Vulpes* in Egypt**. PhD Thesis, Faculty of Education, Ain Shams University, Cairo.  Ferguson W.W. (1981) **The systematic position of *Canis aureus lupaster* (Carnivora: Canidae) and the occurrence of *Canis lupus* in North Africa, Egypt and Sinai.** Mammalia, 45(4): 459–466.  Ferguson W.W. (1981) **The systematic position of *Gazella dorcas* (Artiodactyla: Bovidae) in Israel and Sinai.** Mammalia, 45(4): 453-458.  Flower S.S. (1932) **Notes on the recent mammals of Egypt, with a list of the species recorded from that kingdom**. Proceedings of the Zoological Society of London 1932, 369-450.  Foui E. (2011) **Operation Wallacea: Sinai bats report.** Available at: https://opwall.com/wp-content/uploads/OpWall_Sinai-bat-report_2011.pdf  Fowler H.W. (1914) **Fishes and Reptiles from Assuan, Egypt**. Copeia, 8: 1-2.  Freeman P.W. (1981) **A multivariate study of the family Molossidae (Mammalia, Chiroptera): morphology, ecology, evolution**. Fieldiana Zoology, New Series No. 7, Field Museum of Natural History, Chicago, 173 pp.  Gabriel A.G. & Steven-Corbet A. (1949) **Results of the Armstrong College expedition to Siwa Oasis (Libyan desert), 1395, under the leadership of Prof. J Omer-Cooper.** Bulletin de la Societe Fouad 1er d'Entomologie, 33: 373-9.  Gaisler J., Madkour G. & Pelikan J. (1972) **On the bats of Egypt**. Acta Scientiarum Naturalium Academiae Scientarium Bohemoslovaceae Brno, 6(8):1-40.  Ghazali O.F. & Baha El Din S. (2005) **On the occurrence of *Psammophis punctulatus* Bibron & Dumeril 1854 in Egypt.** Herpetological Bulletin, 94: 4-5.  Ghobashi A.A., Abu Eglah M.H., Tantawy H.M. & Ibrahim A.A. (1990) **Herpetofaunal survey of Al-Arish area (North Sinai) with special reference to their habitat and seasonal distribution**. Proc. Zool. Soc. A.R.E, 21: 273-290.  Gilbert, F. & Zalat, S. (2007). **Butterflies of Egypt: atlas, red data listing & conservation**. Egyptian Environmental Affairs Agency.  Goodman S. M. & Helmy I. (1986) **The sand cat *Felis margarita* Loche, 1858 in Egypt**. Mammalia, 50:120-123.  Graves P. (1915) **A list of the butterflies of Egypt with some notes on those of the Sinai.** Bulletin de la Société entomologiques d'Egypte, 4: 135-157.  Graves P. (1918) **Lampides ethoda, Wlk, and Lycaena itea, Wlk = Lampides thebana, Stdgr., and Azanus ubaldus, Cr.** Entomologist, 51: 98-100.  Graves P. (1918) **Some new forms of Lycaenidae from Egypt.** Entomologist, 51: 97-98.  Harrison D.L. (1961) **On Savi’s Pipistrelle, (*Pipistrellus savii* Bonaparte, 1837) in the Middle East, and a second record of *Nycticeius schlieffeni* Peters 1859 from Egypt**. Senckenbergiana Biologica, 42 (1/2): 41-44.  Harrison D.L. (1964) **The Mammals of Arabia. Vol 1. Introduction, Insectivora, Chiroptera, Primates**. pp 192. Benn, London, 1st edition.  Harrison D.L., Bates P.J.J. (1991) **The mammals of Arabia**. 2nd edition. Harrison Zoological Museum Publication.  Harrison D.L., Makin D. (1988) **Significant new records of Vespertilionid Bats (Chiroptera: Vespertilionidae) from Israel**. Mammalia, 52(4):593–596.  Hart H.C. (1891) **Some account of the flora and fauna of Sinai, Petra, and Wadi 'Arabah.** Committee of the Palestine Exploration Fund, London.  Hassan M.M. & Fadl H.H. (2000) **Contribution to the insect fauna of Gabal Elba and the Red Sea coast**. Bull. Ent. Soc. Egypt, 78: 145-175.  Hayman R.W. (1948) **The Armstrong College Zoological Expedition to Siwa Oasis (Libyan Desert) 1935: Mammalia**. Proceedings of the Egyptian Academy of Sciences, 4:38–42.  Hill J.E. & Harrison D.L. (1987) **The baculum in the Vespertilioninae (Chiroptera: Vespertilionidae) with a systematic review, a synopsis of *Pipistrellus* and *Eptesicus*, and the descriptions of a new genus and subgenus**. Bulletin of the British Museum (Natural History) Zoology, 52:225-305.  Hoogstraal H. & Traub R. (1963) **The fleas (Siphonaptera) of Egypt. Host-parasite relationships of Insectivora and Chiroptera**. Journal of the Egyptian Public Health Association, 38(3):111-130.  Hoogstraal H. (1962) **A brief review of the contemporary land mammals of Egypt (including Sinai). 1. Insectivora and Chiroptera**. Journal of the Egyptian Public Health Association, 37(4):143-162.  Hoogstraal H. (1963) **A brief review of the contemporary land mammals of Egypt (including Sinai). 2. Lagomorpha and Rodentia**. Journal of the Egyptian Public Health Association, 38(1): 1-35.  Hoogstraal H. (1964) **A brief review of the contemporary land mammals of Egypt (including Sinai). 3. Carnivora, Hyracoidea, Perissodactyla and Artiodactyla**. Journal of the Egyptian Public Health Association, 39(1): 205-239.  Ibrahim A.A. (2000) **A radiotelemetric study of the body temperature of *Varanus griseus* (Sauria - Varanidae) in Zaranik Protected Area, North Sinai, Egypt**. Egyptian Journal of Biology, 2: 57-66.  Ibrahim A.A. (2000) **Geographic distribution: *Hemidactyllls flaviviridis***. Herpetological Review 31(3): 185.  Ibrahim A.A. (2000) **Geographic distribution: *Tarentola annularis* annularis**. Herpetological Review 31(3): 185.  Ibrahim A.A. (2001) **A Note on the Diet of the White-spotted gecko, *Tarentola a. annularis***. Gecko, 2: 6.  Ibrahim A.A. (2001) **Geographic distribution: *Bufo regularis***. Herpetological Review 32(2): 112.  Ibrahim A.A. (2001) **Geographic distribution: *Coluber florulentus florulentus***. Herpetological Review, 32(1): 59.  Ibrahim A.A. (2001) **Geographic distribution: *Malpolon monspessulanus insignitus***. Herpetological review: 32(2): 123.  Ibrahim A.A. (2001) **Geographic distribution: *Ptychadena mascareniensis***. Herpetological Review 32(2): 115.  Ibrahim A.A. (2001) **Geographic distribution: *Ptyodactylus h. hasselquistii***. Herpetological Review 32(2): 120.  Ibrahim A.A. (2002) **Activity area, movement patterns, and habitat use of the desert monitor, *Varanus griseus*, in the Zaranik Protected Area, North Sinai, Egypt.** African Journal of Herpetology, 51(1): 35-45.  Ibrahim A.A. (2002) **Activity patterns and microhabitat use of the Starred agama *Laudakia stellio* (Linnaeus, 1758) in AL-Arish city, North Sinai, Egypt**. Egypt. J. zool., 39, 173-196.  Ibrahim A.A. (2002) **Geographic Distribution: *Psammophis sibilans sibilans***. Herpetological Review, 33: 69.  Ibrahim A.A. (2003) **Diet and reproduction of the Indian Leaf-toed gecko, *Hemidactylus flaviviridis* (Sauria: Gekkonidae), in Ismailia governorate, with consideration to its distribution in the Suez Canal area, Egypt**. J. Union Arab Biol. Cairo, 19(A): 125-151.  Ibrahim A.A. (2004) **Behavioural ecology of the White-spotted Gecko, *Tarentola annularis* (Reptilia: Gekkonidae), in Ismailia City, Egypt**. Zoology in the Middle East, 31: 23-38.  Ibrahim A.A. (2007) **Ecology of the Mediterranean Geko, *Hemidactylus turcicus* (Linnaeus, 1785) (Reptilia: Gekkonidae), in North Sinai, Egypt**. Zoology in the Middle East, 41: 41-54.  Ibrahim A.A. (2008) **Amphibians and reptiles of the Suez Canal University campuses, Egypt**. Herpetological Bulletin, 105: 1-9.  Ibrahim A.A. (2012) **New records of the Diced snake (*Natrix tessellata*), in the Suez Canal zone and Sinai**. Amphibian and Reptile Conservation 6(2):2-4(e42).  Ibrahim A.A. (2013) **The Herpetology of the Suez Canal Zone, Egypt.** Vertebrate Zoology, 63(1): 87-110.  Ibrahim A.A., Saleh M.A., Dixon J.R. & Mahmoud I.M. (2000) **On the ecology of the fringe-toed lizard, *Acanthodactylus scutellatus* (Sauria: Lacertidae) in North Sinai, Egypt**. J. Egypt. Ger. Soc. Zool., 32 (A): 335-355.  James M., Gilbert F. & Zalat S. (2003) **Thyme and isolation for the Sinai Baton Blue butterfly (*Pseudophilotes sinaicus*)**. Oecologia, 134: 445-453.  Kamal A., Soliman M. & EL-Assy Y. (1966) **New record of the Amphibians and reptiles of some districts of the western Egyptian desert**. Bulletin de l'instiude du deser d'egypte, 16: 145-157.  Kamal A., Soliman M. & EL-Assy Y. (1966) **New record of the Amphibians and reptiles of two other districts of the western Egyptian desert**. Bulletin de l'instiude du deser d'egypte, 16: 159-167.  Kark S., Warburg I. & Werner Y.L. (1997) **Polymorphism in the snake *Psammophis schokari* on both sides of the desert edge in Israel and Sinai**. Journal of Arid Environments, 37: 513-527.  Kiefer A. (2007) **Phylogeny of Western Palaearctic long-eared bats (Mammalia, Chiroptera, Plecotus): a molecular perspective**. PhD thesis, Johannes Gutenberg-Universität in Mainz.  Kneucker A. (1903) **Zoologische Ausbeute einer botanischer Studienreise durch die Sinai-Halbinsel im März und April 1902**. Verhandlungen der zoologischer-botanischer Gesellschaft im Wien, 53: 575-587.  Kock D. (1969) **Die Fledermaus-Fauna des Sudan**. Abhandlungen der Senckenbergischen Naturforschenden Gesellschaft, Band 521:1:238.  Kock D., Al-Jumaily M. & Nasher A.K. (2002) **Horseshoe bats, genus *Rhinolophus* Lacépède, 1799 (Mammalia: Chiroptera: Rhinolophidae), of Yemen**. Fauna of Arabia, 19:507–515.  Koopman K.F. (1975) **Bats of the Sudan**. Bulletin of the American Museum of Natural History, 154(4).  Lachman E., Carmely H. & Werner Y.L. (2006) **Subspeciation befogged by the ‘‘Seligmann effect’’: the case of *Laudakia stellio* (Reptilia: Sauria: Agamidae) in southern Sinai, Egypt**. Journal of Natural History, 40(19–20): 1259–1284  Larsen T.B. (1990) **The butterflies of Egypt**. Apollo Books, Svenborg, Denmark.  Lewandowski S. & Lewandowski­Krenz K. (2014) **Beitrag zur Lepidopterenfauna von Ägypten (Lepidoptera), Teil 1: Familien Hesperiidae, Pieridae, Lycaenidae, Nymphalidae, Sphingidae, Lasiocampidae; sowie Erebidae: Unterfamilien Lymantriinae und Arctiinae.** Nachrichten des Entomologischen Vereins Apollo, 34: 175-184.  Lučan R.K., Šálek M. (2013) **Observation of successful mobbing of an insectivorous bat, *Taphozous nudiventris* (Emballonuridae), on an avian predator, *Tyto alba* (Tytonidae)**. Mammalia, 77(2):235-236.  Macy R.W., Heyneman D., Kuntz R.E. (1961) **Records of trematodes of the families Lecithodendriidae, Dicrocoeliidae, and Heterophyidae from Chiroptera collected in Egypt and Yemen, S. W. Arabia**. Proc. Helminthol. Soc. Washington, 28:13-17.  Madkour G. (1977) **Further observations on bats (Chiroptera) of Egypt**. Agricultural Research Review, 55(1):173–184.  Madkour G. (1977) ***Rousettus aegyptiacus* (Megachiroptera) as a fruit eating bat in A.R. EGYPT**. Agricultural research review, 55(1):167-172.  Madkour G. (1978) **Significance of the distal part of the humerus in the identification of Egyptian bats**. Zoologischer Anzeiger, 201(5/6):387-390.  Meakin K., de Kort S.R., Gilbert H., Gilbert F., et al. (2005) **Monitoring birds, reptiles and butterflies in the St Katherine Protectorate, Egypt**. Egyptian Journal of Biology, 7: 66-95.  Milto K.D. (2017) **New records of reptiles on the Red Sea Coast, Egypt, with notes on zoogeography.** Russian Journal of Herpetology, 24(1):11-21.  Nader I.A. & Kock D. (1983) **Notes on some bats from the Near East (Mammalia: Chiroptera)**. Zeitschrift für Säugetierkunde, 48:1–9.  Nader I.A. (1982) **New distributional records of bats from the Kingdom of Saudi Arabia (Mammalia: Chiroptera)**. Journal of Zoology, 198(1):69-82.  Nakamura I. & Benyamini D. (1973) ***Euchloe falloui* Allard (Lepidoptera; Pieridae) from Egypt and the Sinai**. Entomologist, 106: 267-8.  Nakamura I. (1975) **Descriptions of two new species of butterflies (Lepidoptera, Lycaenidae) from the South Sinai**. Journal of Entomology (B), 44(3): 283-295.  Nakamura I. (1977) **On the identity of some of the Lycaenidae species (Lep.) described by Francis Walker (1870) from Egypt and the Sinai.** Entomologist's Gazette, 28: 9-14.  Osborn D.J. & Helmy I. (1980) **The contemporary land mammals of Egypt (including Sinai).** Fieldiana Zoology, New Series, No 5: 1-579.  Osborn D.J. (1988) **New bat records from the Red Sea Mountains of Egypt**. Mammalia, 52(4):596-598.  Owen R.D., Qumsiyeh M.B. (1987) **The subspecies problem in the Trident leaf-nosed bat, *Asellia tridens*: homomorphism in widely separated populations**. Zeitschrift Für Saeugetierkunde, 6:329-337.  Qumsiyeh M.B. (1985) **The bats of Egypt**. Special Publication of the Texas Technological University, 23: 1-102.  Qumsiyeh M.B. (1996) **Mammals of the Holy Land**. Texas Tech University Press, Lubbock.  Robbins L.W., Sarich V.M. (1988) **Evolutionary relationships in the family Emballonuridae (Chiroptera)**. Journal of Mammalogy, 69(1):1-13.  Rothschild C. (1901) **Lepidoptera from Egypt and the Soudan**. Novitates Zoologicae, V. 8.  Saber S.S. (1994) **Checklist of the Saurian fauna of Southern Sinai, Egypt**. Al-Azhar Bull. of Science, 5(1): 183-201.  Saber S.S. (1994) **Herpetological survey of Suez province, Egypt**. Bull. Biol. Soc. Egypt, 42.  Saber S.S. (1994) **Ophidian community of Sinai, Egypt**. Al-Azhar Bull. of Science, 5(1): 203-217.  Saleh M.A. & Basuony M.I. (1997) **Ecological distribution of land mammals in South Sinai, Egypt**. Al-Azhar Bulletin of Science, 8(2): 653-670.  Saleh M.A. & Basuony M.I. (1998) **A contribution to the mammalogy of the Sinai Peninsula**. Mammalia, 62(4): 557-575.  Saleh M.A. & Basuony M.I. (2005) **The Zoril, *Ictonyx striatus erythreae* De Winton, 1898 in Egypt**. Egyptian Journal of Biology, 7: 103-107.  Saleh M.A. & Basuony M.I. (2014) **Mammals of the Genus *Canis* Linnaeus, 1758 (Canidae, Carnivora) in Egypt**. Egyptian Journal of Zoology, 62: 49-92.  Saleh M.A. & Basuony M.I. (2014) **New mammalian records from Egypt**. Egyptian Journal of Zoology, 62: 111-130.  Saleh M.A. (1987) **The decline of gazelles in Egypt**. Biological Conservation, 39: 83-95.  Saleh M.A., Basuony M.I., Galhoum A. & Tolba M. (2003) **Biodiversity and zoogeography of the Qattara Depression, Western Desert, Egypt**. Egyptian Journal of Zoology, 40: 357-387.  Saleh M.A., Helmy I. & Giegengack R. (2001) **The cheetah, *Acinonyx jubatus* (Schreber, 1776) in Egypt (Felidae, Acinonychinae)**. Mammalia 65: 177-194.  Sanborn C.C. & Hoogstraal H. (1955) **The identification of Egyptian bats**. Journal of the Egyptian Public Health Association, 30(4):103-119.  Saoud M.F. & Ramadan M.M. (1977): **On a new trematode, *Prohemistomum azimi* n. sp. (Trematoda: Cyathocotylidae) from the Egyptian slit-faced bat**. Zeitschrift für Parasitenkunde, 53(3):281–285.  Sayed M.T., Rostom Z.M.F. & Kaschef A.H. (1964) **Contributions to the insect fauna of some oases of the Egyptian Western Desert.** Bulletin de la Société entomologiques d'Egypte, 48: 259-267.  Schmidt K.P. & Marx H. (1957) **Results of the NAMRU-3 southeastern Egypt expedition, 1954. 2. Reptiles and amphibians**. The Zoological Society of Egypt Bulletin, 13:16–28.  Schwann H. (1905) **A list of the mammals collected by the Hon. N. C. Rothschild, the Hon. F. R. Henley, and Mr. A. F. R. Wollaston in Egypt and the Soudan in January, February, and March 1904**. Novitates Zoologicae, 12:1–5.  Semida F.M. (1985) **Survey and ecological studies of non-true pollinators of southern Sinai.** MSc Thesis, Suez Canal University, Ismailia.  Setzer H.W. (1952) **Notes on mammals from the Nile Delta region of Egypt**. Proceedings of the United States National Museum, 102(3305):343–369.  Setzer H.W. (1955) **Two new Jerboas from Egypt**. Proceedings of The Biological Society of Washington, 68: 183–184.  Setzer H.W. (1957) **The hedgehogs and shrews (Insectivora) of Egypt**. Journal of the Egyptian Public Health Association, 32(1): 1-17.  Setzer H.W. (1958) **The gerbils of Egypt**. The Journal of the Egyptian Public Health Association, 23(6): 205-227.  Setzer H.W. (1958) **The Jerboas of Egypt**. Journal of the Egyptian Public Health Association, 33(3): 87-94.  Setzer H.W. (1958) **The Mustelids of Egypt**. The Journal of the Egyptian Public Health Association, 23(6): 199–204.  Setzer H.W. (1959) **The spiny mice (*Acomys*) of Egypt**. The Journal of the Egyptian Public Health Association, 34(3): 93-101.  Setzer H.W. (1961) **The canids (Mammalia) of Egypt**. Journal of the Egyptian Public Health Association, 36(3): 113-118.  Setzer H.W. (1961) **The jirds (Mammalia: Rodentia) of Egypt**. Journal of the Egyptian Public Health Association, 36(3): 81-92.  Setzer H.W. (1963) **Notes on some Egyptian rodents**. Journal of the Egyptian Public Health Association, 38(2): 51-60.  Shahin A.A. & Ata A.T. (2004) **C-banding Karyotype and Relationship of the Dipodids *Allactaga* and *Jaculus* (Mammalia: Rodentia) in Egypt**. Folia biologica (Kraków), 52 (1-2): 25-31.  Shehab A., Mamkhair I., Amr Z. (2006) **First record of the Lesser Horseshoe bat, *Rhinolophus hipposideros* (Bechstein, 1800) (Rhinolophidae, Chiroptera) from Syria**. Hystrix, the Italian Journal of Mammalogy, 17(2):161-166.  Shifman S., Shacham B. & Werner Y.L. (1999) ***Tropioclotes nattereri* (Reptilia, Gekkonidae) - comments on validity, variation and distribution**. Zoology in the Middle East, 17: 51-66  Sohail S, Sameeh A.M. & Kareem M.S. (2014) **Population studies on two sympatric bat species: *Taphozous Perforatus* and *Rhinopoma Hardwickii* (Chiroptera: Microchiroptera) from Egypt**. Egyptian Journal of Zoology, 64: 13–31.  Soliman S. & Mohallal E. (2009) **Patterns of reproduction in two sympatric gerbil species in arid Egypt**. Integrative Zoology, 4(2):248-253.  Soliman S. & Mohallal E. (2014) **A survey of the mammalian fauna of Siwa Oasis, Egypt**. Egyptian Journal of Zoology, 61:171–186.  Soultan A., Attum O., Hamada A., et al. (2017) **Recent observation for leopard *Panthera pardus* in Egypt**. Mammalia, 81(1): 115–117.  Tawfik A. (1994) **Taxonomic analysis of two chamaeleon species (Chamaeleontidae, Reptilia): morphological and biochemical studies**. J. Egypt. Ger. Soc. Zool., 15(A): 191-203.  Tolba M., Basuony M.I. & Khedr A. (2004) **Ecosystem structure and sensitivity modeling of Hamata area, Eastern Desert, Egypt using GIS**. Al-Azhar Bulletin of Science, 15(1): 55-82.  Tregenza L. (2004) **The Red Sea Mountains of Egypt and Egyptian Years**. AUC Press, Cairo.  Van Cakenberghe V., De Vree F. (1998) **Systematics of African *Nycteris* (Mammalia: Chiroptera) Part III. The *Nyteris thebaica* group**. Bonner Zoologische Beitrage, 48(2):123-166.  Wacher T., Baha El Din S., Mikhail G. & Baha el Din M. (2002) **New observations of the 'extinct' Barbary sheep *Ammotragus lervia ornata* in Egypt**. Oryx, 36(3): 301-304  Wagner P. & Böhme W. (2006) **A new species of the genus *Trapelus* Cuvier, 1816 (Squamata - Agamidae) from arid central Africa**. Bonner Zool. Beitr. 55(2): 81-87.  Wahrman J. & Gourevitz P. (1973) **Extreme chromosome variability in a colonising rodent.** Chromosomes Today, 4: 399-424.  Wallach V. (1999) **Geographic distribution: *Ramphotyphlops braminus* (Brahminy Blind Snake)**. Herpetological Review 30(4): 236.  Wassif K. & Hoogstraal H. (1953) **The Mammals of South Sinai, Egypt**. Proceedings of the Egyptian Academy of Science, 9: 63-79.  Wassif K. & Soliman S. (1980) **Population studies on gerbils of the Western Desert of Egypt, with special reference to *Gerbillus andersoni*, de Winton**. Proceedings of the 9^th^ Vertebrate Pest Conference, pp.154–160.  Wassif K. (1944) **On the occurrence of *Paraechinus dorsalis* (Anderson and De Winton) in South Sinai, with a note on the osteology of the animal**. Bull. Fac. Sci. Egypt. Univ. 25: 203-211.  Wassif K. (1954) **On a collection of mammals from northern Sinai**. Bulletin de l'Institute du Désert de l'Egypte, 3(1): 107-118.  Wassif K. (1954) **On the occurrence of hedgehogs of the genus *Paraechinus* in the El Tahreer Province of Egypt**. Bull. Zool. Soc. Egypt 11: 40-47.  Wassif K. (1954) **The Bushy-Tailed Gerbil, *Gerbillus calurus* Thomas, of South Sinai**. Journal of Mammalogy, 35(2):243.  Wassif K., Lutfy R.G. & Wassif S. (1969) **Morphological, cytological and taxonomic studies of the rodent genera *Gerbillus* and *Dipodillus* from Egypt**. Proc. Egypt. Acad. Sci., 22: 22:77.  Wassif K., Madkour G. & Soliman S. (1984) **Fauna and Flora of Egypt. 1. On a collection of bats from Egypt**. Academy of Scientific Research and Technology, Natural History Museum of Egypt, 2: 1-36.  Werner Y.L. & Ashkenazi S. (2010) **Notes on some Egyptian Lacertidae, including a new subspecies of *Mesalina*, involving the Seligmann effect**. Turk J Zool, 34: 123-133.  Werner Y.L. (1968) **Distribution of the Saharan *Sphenops sepsoides* (Reptilia: Scincidae) in Israel and Jordan**. Herpetologica, 24(3): 238-242.  Werner Y.L. (1983) **Lizards and snakes from eastern Lower Egypt in the Hebrew University of Jerusalem and Tel Aviv University with range extensions**. Herpetological Review, 14(1), 29-31.  White M.L., Dauphiné N.S., Mohamed A.E. (2007) **Recent surveys and comparisons of birds and reptiles in St. Katherine Protectorate, Egypt, 2007**. Operation Wallacea/BioMAP Report.  Williams C.B. & Bishara I. (1929) **The seasonal abundance of four common butterflies in Egypt**. Bulletin de la Société Royale entomologiques d'Egypte, 13: 85-92  Williams G.H. (1917) **Notes on some Egyptian butterflies.** Bulletin de la Société entomologiques d'Egypte, 5: 91-4.  Wiltshire E.P. (1948) **The Lepidoptera of the Kingdom of Egypt.** Bulletin de la Societe Fouad 1er d'Entomologie, 32: 203-294.  Yassen A.E., Hassan H.A., Kawashti L.S. (1994) **Comparative study of the karyotypes of two Egyptian species of bats, *Taphozous perforatus* and *Taphozous nudiventris* (Chiroptera: Mammalia)**. Experientia, 50(11):111-1114. |
| --- |
